# Nucleolar asymmetry and the importance of septin integrity upon cell cycle arrest

**DOI:** 10.1371/journal.pone.0174306

**Published:** 2017-03-24

**Authors:** Urvashi Rai, Fadi Najm, Alan M. Tartakoff

**Affiliations:** 1 Cell Biology Program/Department of Molecular and Microbiology, Case Western Reserve University, Cleveland, Ohio, United States of America; 2 Division of Medical Sciences, Harvard Medical School, Boston, Massachusetts, United States of America; 3 Department of Pathology, Case Western Reserve University, Cleveland, Ohio, United States of America; Institut de Genetique et Developpement de Rennes, FRANCE

## Abstract

Cell cycle arrest can be imposed by inactivating the anaphase promoting complex (APC). In *S*. *cerevisiae* this arrest has been reported to stabilize a metaphase-like intermediate in which the nuclear envelope spans the bud neck, while chromatin repeatedly translocates between the mother and bud domains. The present investigation was undertaken to learn how other features of nuclear organization are affected upon depletion of the APC activator, Cdc20. We observe that the spindle pole bodies and the spindle repeatedly translocate across the narrow orifice at the level of the neck. Nevertheless, we find that the nucleolus (organized around rDNA repeats on the long right arm of chromosome XII) remains in the mother domain, marking the polarity of the nucleus. Accordingly, chromosome XII is polarized: TelXIIR remains in the mother domain and its centromere is predominantly located in the bud domain. In order to learn why the nucleolus remains in the mother domain, we studied the impact of inhibiting rRNA synthesis in arrested cells. We observed that this fragments the nucleolus and that these fragments entered the bud domain. Taken together with earlier observations, the restriction of the nucleolus to the mother domain therefore can be attributed to its massive structure. We also observed that inactivation of septins allowed arrested cells to complete the cell cycle, that the alternative APC activator, Cdh1, was required for completion of the cell cycle and that induction of Cdh1 itself caused arrested cells to progress to the end of the cell cycle.

## Introduction

Upon loss of the activator of the anaphase promoting complex, Cdc20, in *S*. *cerevisiae*, DNA replicates and the bud grows until it is essentially as large as the mother. The metaphase-like arrest at this point is known as “medial nuclear division arrest.” Cohesin keeps sister chromatids associated with each other and the spindle does not elongate. At this point, the cell is poised to enter into anaphase [1–7].

By contrast to arrest imposed by treatment with nocodazole (to depolymerize microtubules) or hydroxyurea (to inhibit DNA synthesis), upon APC inactivation by Cdc20 depletion the nucleus spans the bud neck (medial nuclear division arrest: MND), and the chromatin mass undergoes irregular “transits” between the mother and bud every several minutes [8, 9]. This behavior was first detected by staining the DNA of living cells and observing them under u.v. illumination. Transits can also be provoked by DNA damage, e.g. upon chromosome breakage when more than one centromere is active [10]. Transits are silenced by addition of nocodazole or destabilization of cytoplasmic microtubules in *tub2-401* [8, 9]. They thus appear to result from the opposed pulling by cytoplasmic microtubules in both mother and bud domains, which is punctuated by their dynamic instability. It is striking that chromatin can continue to transit for hours.

APC/Cdc20 activation leads to cohesin cleavage, and triggers spindle elongation. By late anaphase, the key APC activator is the Cdc20-related protein, Cdh1/Hct1, that confers a distinct but overlapping specificity upon the APC, thereby completing the cell cycle [11–13].

During the normal cell cycle, an hourglass-shaped assembly of septins encircles the bud neck, where both the ER and the cell cortex have a distinct composition [14–17]. The hourglass transforms into a pair of parallel rings just prior to cytokinesis and then disassembles. These events are roughly coincident with activation of Cdh1. Photobleaching studies show that septins restrict diffusion of proteins of the cell cortex and membrane proteins of the ER between the mother and bud [18, 19].

Our present goal is to identify requirements for maintaining cell cycle arrest upon Cdc20 inactivation. Since the classic studies of chromatin transits were based on protocols in which APC temperature-sensitive mutant cells were arrested at 36°C and observed after return to room temperature for 15–30 minutes, we have first reexamined basic features of arrest under circumstances in which temperature shifts are not involved (i.e. the position of chromatin, the spindle and the nuclear envelope). This is achieved using strains in which Cdc20 expression is under control of a methionine-repressible promoter. We then focus on the question of why the nucleolus does not participate in transits and conclude that passage of the genome into the bud domain can be subject to chromosome-specific controls. Moreover, the integrity of septins at the bud neck is critical for maintaining arrest.

## Materials and methods

### Growth conditions and reagents

Yeast strains were grown in synthetic media supplemented with 2% glucose and 20 mg/L adenine [20]. Methionine-free media were used to grow cells that carry the *MET3-CDC20* cassette. Unless otherwise indicated, all procedures were conducted at 23°C.

Standard chemicals were from Sigma/Aldrich. Other chemicals were obtained from the following sources: alpha factor peptide (Cleveland Clinic Core Laboratory, Cleveland, OH), DiOC6 (Thermo Fisher Scientific), FM4-64 (Thermo Fisher Scientific), G418 (Amresco), latrunculin A (AdipoGen Life Sciences), myriocin (Sigma-Aldrich), nocodazole (Sigma/Aldrich), nourseothricin (Jena Bioscience), rhodamine-Concanavalin A (Vector Laboratories).

### Yeast strain constructions

The cells used in this study were from either the S288C or W303 background and are listed in S1 Table. In strains expressing GFP-tagged proteins, the tags were at the C-terminus, except for GFP-Cdc3, GFP-Rap1, Nup49-GFP (internal tag) and Cse4-GFP (see text). The C-terminally-tagged strains were from Invitrogen. Most strains were constructed using classical genetic crosses and sporulation. To delete *LTE1*, a nourseothricin-resistance module was copied by PCR using primers that included flanking sequences upstream and downstream of *LTE1*. Drug-resistant transformants were screened by colony PCR. The *MET3-CDC20* cassette was introduced into cells by transformation with the integrating plasmids (pAT1519, pAT1520) that had been linearized with Msc1. Transformants were screened for arrest upon transfer to methionine-containing medium for 4 hours. We observed that >80% of cells had buds whose diameter was comparable to the diameter of the maternal lobe by this time. S2 Table lists plasmids that were used in this study.

Petite strains were generated by overnight growth in the presence of ethidium bromide at neutral pH and were then were screened for lack of growth on glycerol.

### Cell cycle arrest

To achieve arrest, the APC activator, Cdc20, was under control of a methionine-repressible promoter (*MET3*). In general, log-phase cycling cells in methionine-free medium were transferred to methionine-containing medium (20 mg/L methionine) with 2% glucose and incubated for 3–4 hours at 23°C until >80% of cells arrested, as judged by their bud size and by monitoring the distribution of fluorescent histone signal across the bud neck [6].

To estimate the stability and escape from arrests, we always counted more than 200 cells in three or more experiments.

### Live cell imaging

Cells were sedimented in a table-top centrifuge and 1.5 microliter samples of the pellet were applied to 1.5% agarose pads prepared in appropriate medium. They were then overlaid with a coverslip that was sealed with Vaseline [21] and were examined in a Deltavision RT epifluorescence microscope with an automated stage (Applied Precision, Inc). The microscope was equipped with a 100x oil immersion objective (Olympus UPlanApo 100x/1.40; 0.17/FN26.5) and images were recorded without binning. The Microscope uses an Insight Solid State Illuminator 7-color combined unit to deliver the specified excitation bandwidth in combination with an optimized polychroic bandpass filter (Semrock #34-100608-001 Rev A) and appropriate emission filters. The relevant filters have the following characteristics: DAPI (390/18, 435/48), GFP (475/28, 523/36) and TRITC (542/27, 594/45), CFP (438/24, 465/30). z-stacks of 7–14 images were captured at 0.2–1 micron intervals using a CCD digital camera (Photometrics CoolSnap HQ). Out-of-focus light was digitally removed using the Softworks deconvolution software (Applied Precision, Inc). Images were exported as .tif files and figures were composed using Adobe Photoshop CS (Adobe, Inc). Images are often presented with pseudocolors in order to heighten color contrast. Brightfield images are often in blue.

More than 100 cells were observed for each condition and the selected illustrations are representative of the large majority of cells (> 80%). Complete through-focal series were examined in all cases. As indicated, most of the illustrations are of projected image stacks.

### Staining of living cells

#### Vacuoles

Cells were arrested for 3 hours and then stained by resuspension in 20 micromolar FM4-64 in CSM-glucose medium containing 0.5% DMSO and incubated for 1 hour at 23°C. The cells were then examined using the same microscope settings as for GFP.

#### Lipids

One microliter of a 10 mg/ml DiOC6 stock in DMSO was diluted 1000x into growth medium. Petite cells resuspended in this medium were incubated in the dark for 5 minutes and examined using the same microscope settings as for GFP.

#### Cell wall

To identify the mother *vs* bud cortex, we prestained cycling cells with rhodamine-Con A (50 microgram/ml) in growth medium, 15 minutes ice). After two washes in growth medium, they were recultured in methionine-containing medium for 4 hours. Arrested cells with a stained and an unstained near-circular perimeter on opposite sides of the bud neck were examined to localize Gar1-GFP.

### DNA stain

To stain DNA, growing cells were fixed for 5 minutes by adding formaldehyde at a final concentration of 2%. They were then washed in water, incubated for 5 minutes in 10 micrograms/ml Hoechst 33342, washed with water and examined using a DAPI filter.

### Spindle length calculation

To measure spindle length, arrested cells were imaged at 2-minute intervals for 15 minutes. 0.5 micron sections were collected and spindle length was calculated from the sections in which both extremities exhibited the brightest fluorescence. The average length was calculated for each cell over 15 minutes and the highest percentage change in spindle length was tabulated for each cell. Length was quantitated in a total of 48 cells.

### Statistics

Statistical significance was evaluated by Student’s *t*-test. Primary data tables are included at the end of all other supplementary data.

## Results

### 1. Organization of the nucleus upon arrest

#### Chromatin and the spindle translocate while the nuclear envelope spans the bud neck

**Fig 1:**
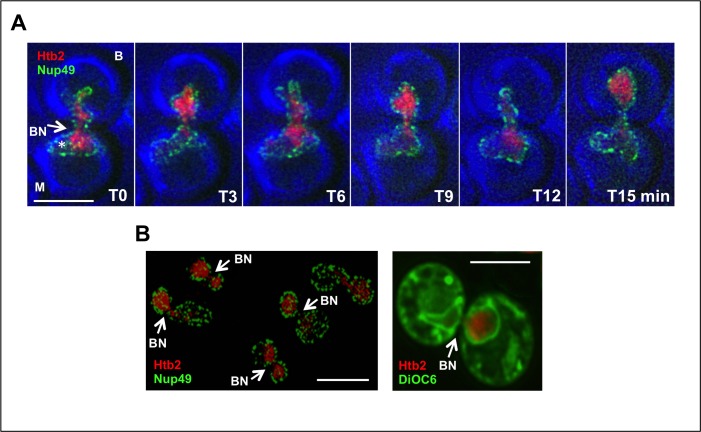
Organization of the Nuclear Envelope in Arrested Cells. (A) Dynamics of the nuclear envelope: Time-lapse images of a *MET3-CDC20* strain expressing Htb2-mRFP and Nup49-GFP, after addition of methionine for 4 hours to deplete Cdc20. Note that the NE extends approximately equally into the mother and bud at all time points. The star (*) indicates a region of the nucleoplasm that is occupied by the nucleolus, judging from examination of other arrested strains. M and B designate the mother and bud domains respectively. In these and all other images, the scale bar is 5 microns. Unless indicated to the contrary, all images are projections of 5–10 Z-sections. In this figure and all others, the bud neck is designated as BN. Strain: ATY4435. (B) The bud neck: Distribution of Nup49-GFP. A *MET3-CDC20* strain expressing Htb2-mRFP and Nup49-GFP was arrested for 4 hr. The arrows indicate the frequent absence of Nup49-GFP from the bud neck. Strain: ATY4435. Exclusion of DiOC6 from the bud neck. A petite *MET3-CDC20* strain expressing Htb2-mRFP was arrested and stained with DiOC6 to detect lipids of the ER (and possibly other membranes [17]). Note the near-absence of signal at the bud neck. This image is from a single 0.5 micron Z-section. Strain: ATY4435.

After depletion of Cdc20 over 4 hours at 23°C, cells are poised to advance into anaphase and to complete the cell cycle. In arrested cells that expressed the nucleoporin, Nup49-GFP, we observed that the nuclear envelope (NE) spanned the bud neck and that the shape of the NE changed during transits (Fig 1A), basically in agreement with electron microscopic studies of the APC ts strain, *cdc16-1* [9]. Specifically, when the chromatin mass (detected by expression of the tagged histone, Htb2-mRFP) was in one parental domain, the NE surrounded it closely, but when chromatin had transited into the other compartment, the NE in the vacated domain had a collapsed appearance. There was no indication that most nuclear pore complexes or the NE moved along with chromatin. Thus, any interactions of chromatin with pores or with the inner membrane could be lost, or must be repeatedly relinquished and reformed during transits [22].

A notable feature of arrest is that the nucleus does not slide back-and-forth across the bud neck. It thus seems likely that the midpoint of the NE is linked to the cell cortex at the level of the bud neck. We therefore asked whether the NE of arrested cells exhibits any special characteristics in this region. Indeed, as for the bud neck of cycling cells [17], both the tagged nucleoporin, Nup49-GFP, and staining with the lipophilic dye, DiOC6, were generally missing at this level (Fig 1B).

**Fig 2:**
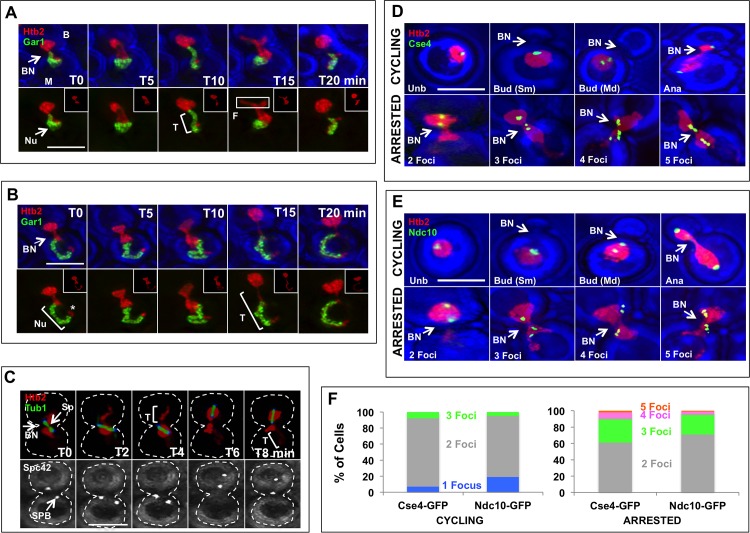
Dynamics of Chromatin and the Nucleolus in Arrested Cells. (A) Time-lapse images of chromatin and the nucleolus. A *MET3-CDC20* strain expressing the tagged histone, Htb2-mRFP, and the tagged nucleolar protein, Gar1-GFP, was arrested by addition of methionine for 4 hours before imaging. Note that the chromatin mass (red) shifted between the mother (M) and bud (B) domains repeatedly and changed shape in doing so. When most of the chromatin was in the bud domain, a narrow tail (T) extended across the mother domain. When the chromatin was retracting from the bud domain, the trailing edge often had a flattened surface (F) (rectangle). The nucleolus (Nu—green) always remained in the mother domain. In the inserts, the green signal is absent so that the red can be more uniformly detected. The blue images are bright-field. Strain: ATY3175. (B) As in Fig 2A except that the nucleolus and chromatin in the mother domain were further extended. In this situation, the tail in the mother domain defined a narrow ribbon that ends in a red patch (*) at its extremity. Strain: ATY3175. (C) Time-lapse images of chromatin, the spindle and the spindle pole body. A *MET3-CDC20* strain expressing Htb2-mRFP, Tub1-GFP and the SPB protein, Spc42-CFP, was arrested by addition of methionine for 4 hours and then imaged. The orientation of the spindle (Sp) shifted and the entire spindle frequently moved from one domain to the other along with chromatin. As shown in the lower images, the SPBs (arrow) remained associated with both extremities of the spindle. Strain: ATY7135. (D-E) Localization of centromeres (D) and kinetochores (E). The tagged variant histone, Cse4-GFP, and the tagged kinetochore protein, Ndc10-GFP, were localized in cycling cells (upper panels) and in a *MET3-CDC20* strain after arrest for 4 hours by addition of methionine (lower panels). Cycling cells: In most unbudded cells (Unb), both Cse4-GFP and Ndc10-GFP formed single clusters, while they had doubled in cells with small buds (Sm) or medium-sized buds (Md) and in cells in anaphase (Ana). Arrested cells: Note the frequent presence of additional fluorescent foci (fragments) for both Cse4-GFP and Ndc10-GFP. Both cells expressing Cse4-GFP with the tag at an internal position and cells with the tag at the C-terminus exhibited comparable fragmentation. Strains: ATY6196, ATY6882, ATY6833. (F) Quantitation of the number of foci detected in preparations equivalent to Fig 2D and 2E. Strains: ATY6196 and ATY6882.

In this situation, time-lapse studies showed that the chromatin mass was highly dynamic and apparently malleable during transits, becoming especially narrowed when traversing the bud neck (Fig 2A and 2B). In the extremes, the chromatin formed either (i) A mass in the bud domain with an extension that reached to the extremity of the mother domain, or (ii) A mass that was largely within the mother domain and often exhibited a concave surface in the bud. Judging from time-lapse series, the flattened or concave surface occurred when the chromatin was retracting from the bud domain. An extensive shift of the chromatin mass occurred every 5–15 minutes. Some transits were complete, whereas in others the chromatin moved only part way across the neck, paused for as long as 20 minutes, and then returned to its compartment of origin. The spanning intermediate was of particular interest because one might expect the medial constriction to impede passage of chromatin across the neck passage (see below).

During arrest, the spindle transected the chromatin mass in 77% of cells (n = 94) (Fig 2C). In the remaining cells, less extensive contact with chromatin was also evident. In accord with the position of chromatin–and presumably due to pulling by cytoplasmic microtubules—the orientation and position of the spindle often changed abruptly in less than 1–2 minutes. Thus, during a typical 15 minute observation period, ~ 38% of cells showed complete spindle displacement between domains, ~ 49% showed displacement of one end to the other domain, and the remaining cells showed essentially no change. As a result, at a given moment the two ends of the spindle were located either in one domain (26% ± 6.4% of cells), on opposite sides of the bud neck (35% ± 12% of cells), or with one extremity contacting the bud neck (39% ± 5.7% of cells, n = 200). Upon arrest, the spindle pole bodies (SPBs) remained at the ends of the spindle (Fig 2C). Because SPBs are embedded in the NE—and since the lateral diffusion of certain proteins of the cell cortex and ER is restricted at the level of the neck–the seemingly facile translocation of the SPBs across the bud neck is notable.

Given the frequency of translocation of chromatin and the apparent constriction at the level of the bud neck, we evaluated some more specific features of chromosome organization that have not previously been studied. The following sections therefore concern the position of centromeres, kinetochores, telomeres, the nucleolus, and the orientation of chromosome XII in arrested cells.

#### Fragmentation of clusters of centromeres and kinetochores

Centromeres cluster adjacent to the SPB during essentially the entire cell cycle [23]. We have localized centromere clusters by expressing the tagged variant histone, Cse4-GFP, and kinetochores by localizing the tagged inner kinetochore protein, Ndc10-GFP. Among cycling cells, ~ 13% (7–19%, n = 60–75) had a single cluster of each marker, as expected at the beginning of the cell cycle, while ~ 87% had a pair of clusters of each marker (Fig 2D–2F) [24]. Only a limited number of cells (~ 6.5%) had fluorescent foci that were separate from the clusters.

After arrest for 4 hours, although the majority of cells (61–71%, n = 84–100) had two clusters, 29–39% of cells showed evidence of fragmentation of these markers (Fig 2D–2F). Equivalent distributions were seen in fixed preparations, thereby ensuring that they did not result from motion during imaging. Both the clusters and the fragmentation could be found in either the mother or bud domain. The fragmentation could result from the force exerted on the SPBs by cytoplasmic microtubules. In support of this hypothesis, we found that modest fragmentation of both markers also occurred when a ts SPB *spc42-10* mutant was shifted to the restrictive temperature (S1 Fig).

Time-lapse imaging showed only minor changes in overall length of the spindle once cells had been arrested (maximum 12.7 ± 8.1% change over 15 minutes, n = 48).

In sum, forces exerted on SPBs of arrested cells appear to be sufficient to affect the clustering of centromeres.

#### The nucleolus and chromosome XII define a polarity axis for arrested cells

**Fig 3:**
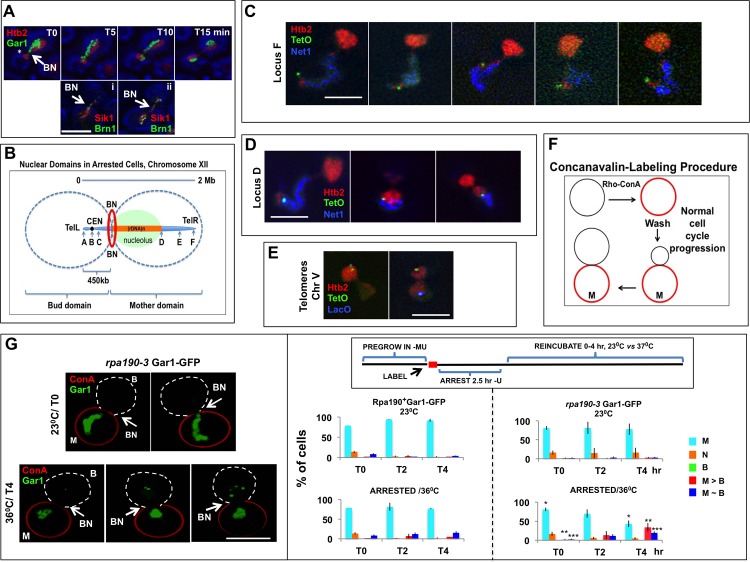
Polarity of Chromosome XII and the Nucleolus. (A) Distribution and coherence of the nucleolus. The top panels illustrate the relative behavior of a nucleolar protein (Gar1-GFP) and Htb2-mRFP during entry into anaphase of a normal cell cycle. Note that a limited amount of chromatin emerged into the bud domain at an early time-point (*) after being constricted at the level of the bud neck. The nucleolar signal then follows slowly, remaining in contact with the stretched chromatin mass. It subsequently is resolved into two equal parts that associate with the chromatin masses. Strain: ATY2416. The lower panels illustrate an intermediate time point when two nucleolar markers can be seen to traverse the neck during the normal cell cycle. These are the condensin, (Brn1-GFP) (that coats compacted rDNA and defines a narrow thread), and Sik1-mRFP, that occupies a broader territory. Both markers are compressed as the nucleolus traverses the bud neck region. Strain: ATY7473. (B) Schematic representation of nuclear domains and chromosome XII in arrested cells. The diagram illustrates the anticipated position of chromosome XII when the nucleolus (green) abuts on the bud neck. The position of the septin ring is also indicated, suggesting that this ring—in conjunction with the nucleolus—could control passage across the neck. The lacO or tetO loci that we have followed are from the following strains: locus A (ATY7633), locus B (ATY7634), locus C (ATY7733), locus D (ATY7241), locus E (ATY7637) and locus F (ATY7102). (C) Distribution and organization of telomere XII after arrest. Images of a *MET3-CDC20* strain that expresses Htb2-mRFP, Net1-CFP and a tetO tag at locus F (TelR). In the arrested cells, the tail portion of the chromatin and Net1-CFP (blue) were in the mother domain, whereas the mass of chromatin was in the bud domain. Note that when the tail was fully extended, the red extremity and the tetO locus were distal to Net1-CFP. The portion of the Htb2-mRFP signal corresponding to the nucleolus was relatively faint, perhaps because it is actively transcribed [36]. Strain: ATY7102. (D) Distribution of rDNA: Images of a *MET3-CDC20* strain that expresses Htb2-mRFP, Net1-CFP and a tetO tag at locus D. Cells were arrested by addition of methionine for 4 hours and then imaged. Note that, by contrast to cells with a tagged telomere, the tagged locus abutted on the Net1-CFP signal and that—when the tail was maximally extended—this locus was not within the red telomeric region. Strain: ATY7241. (E) Distribution and organization of the telomeres of chromosome V: A *MET3-CDC20* strain expressing Htb2-mRFP, TelVL-tetO and TelVR-lacO along with YFP-tetR and CFP-lacI was arrested by addition of methionine for 4 hours. In some cells, both TelVL-YFP and TelVR-CFP were located in a single domain (left), while in other cells, the signals were each in a different domain (right). Strain: ATY7272. (F) Concanavalin staining of cells: Cells were stained with rhodamine-conjugated ConA for 15 minutes on ice. The cells were then washed and reincubated. The diagram indicates the staining pattern that was observed before and after reincubation to allow bud growth. (G) Localization of Gar1-GFP in *rpa190-3* ts mutant cells: Images on the left: A *MET3-CDC20 rpa190-3* strain expressing Gar1-GFP was labeled with rho-Con A and arrested by addition of methionine at 23°C. The cells were then shifted to 36°C (or not) and studied after 0–4 hours. The two upper images show that, before reincubation, Gar1-GFP remained in the mother domain (encircled by red) and reached as far as the bud neck. The three lower images are examples in which Gar1-GFP was also detected in the bud domain after a 4 hour reincubation. The bar graphs on the right quantitate the distribution of Gar1-GFP in the same ts cells and also in isogenic Ts+ cells. Note the stability of arrest in the Ts+ cells at both temperatures and the progressive loss of arrest in the ts cells after incubation at 36°C. The distribution of Gar1-GFP is symbolized as follows: M: only in the mother domain, N: contacts the neck or spans the neck, B: only in the bud, M > B: in both domains with more signal in the mother domain, M ~ B: approximately equal in both domains. In this figure and all others that quantitate distribution of markers, the data are given ± one standard deviation. Statistical significance was evaluated by Student’s *t*-test. In the lower right panel, pairs of bars (T0 hr, T4 hr) bearing the same number of stars have been compared to each other. *P < 0.005, **P < 0.05, and ***P < 0.05. Strains: ATY7760 and ATY3175. Parallel studies of *rpa190-3* cells that express a tagged histone (Htb2-GFP) did not show any indication of progression to or beyond anaphase.

The yeast nucleolus is organized around the ~ 1Mb repetitive rDNA locus near the middle of the long right arm of chromosome XII. It is composed of contiguous subcompartments that collectively generate ribosomal subunits [25–28]. The distribution of yeast nucleolar proteins and rDNA changes dramatically during the cell cycle, finally forming a compact spiral structure as cells enter anaphase [29–35]. Visualization of condensins (that associate closely with rDNA), emphasizes that translocation of rDNA is in fact a linear process in which chromosome XII is threaded through the bud neck (Fig 3A, lower panels) (see [29, 34] and the supplementary movie No. 2 in [33]). This chromosome is presumably being pulled by one SPB, although the final phase does not require spindle function [31].

During anaphase of the normal cell cycle, chromatin enters the bud before nucleolar markers begin to enter the bud. In this process, the nucleolus seems to stretch along the surface of chromatin [29, 37, 38]. Although there has been little mention of the site at which the nucleolus is paused, delay can be seen to occur at the level of the bud neck, (Fig 3A, upper panels). The observation that chromatin translocates early could reflect its quite distinct flexible polymer “beads-on-a-string” organization, the modest size of most of its individual transcriptional domains, and the repeated nucleosome disassembly that accompanies transcriptional activation [39–41].

In time-lapse observations of cells that were just transferred to methionine-containing medium to deplete Cdc20, we observed that the nucleolus (tagged with Gar1-GFP–or Bnr1-GFP, Net1-GFP or Sik1-mRFP) remained in the mother domain until well after the size of the bud approximated the size of the mother (S2 Fig and see below). One end of the nucleolus often contacted the bud neck, but it did not remain associated with the neck (Fig 3A–3D). The restriction of the nucleolus that we observed in arrested cells is thus reminiscent of its delayed entry into the bud during the normal cell cycle. We therefore have looked more closely into the organization of chromosome XII and reasons for restriction of the nucleolus (Fig 3B).

In arrested cells in which chromatin was primarily in the bud domain and only a narrow chromatin tail remained in the mother domain, the nucleolar marker, Net1-CFP, aligned along the middle portion of the tail. The distribution of Net1-CFP thus approximated that expected for rDNA, being absent from the extremity of the tail where there was a patch of Htb2-mRFP (stars in Fig 3C).

To learn whether chromosome XII assumes a polarized orientation, we followed a set of chromosomal loci that were tagged with lacO or tetO repeats (loci A-F in Fig 3B) in arrested cells. This chromosome is acrocentric, with only ~ 7% of its length constituting the short left arm. We observed that a tagged locus (locus F) 20 kb from TelXIIR (and loci D and E) was always in the mother domain (n ~ 66). When the nucleolus was maximally extended, locus F marked the terminal patch of Htb2-mRFP (Fig 3C) (n = 35), beyond the Net1-CFP-positive region. When the tag was instead immediately distal to rDNA (locus D), the signal was distal to Net1-CFP yet proximal to the extremity of the tail (Fig 3D). Taken together, these observations are consistent with earlier studies of arrested cells [42]; however, the earlier studies did not distinguish mother *vs* bud domains. As is explained below, we can make this distinction either by time-lapse imaging or by prelabeling the cortex of the mother with fluorescent ConA.

By contrast to TelXIIR, we found that TelXIIL (locus A), CENXII (locus B), and locus C (slightly distal to CENXII) were in the bud domain in 67–70% of arrested cells (n ~ 70). The diagram in Fig 3B therefore approximates the situation when chromatin has been maximally pulled toward the bud and the nucleolus abuts on the bud neck. Because yeast centromeres cluster together, any restrictions of localization of CENXII seem likely to influence the distribution of all centromeres.

#### Telomere distribution in arrested cells

During the mitotic cell cycle, telomeres form 3–8 dynamic clusters at the periphery of the nucleus [24, 43–45]. In arrested cells, examination of through-focal series showed that the tagged telomere-associated protein, GFP-Rap1, was concentrated in 3–8 foci that were located in either the mother or bud domains (n = 60). As might be expected due to the ongoing transits, the foci were less often concentrated at the margin of chromatin than in cycling cells (S3 Fig). Moreover, judging from the localization of lacO and tetO repeats at both ends of chromosome V, these specific telomeres could be found at the surface of the chromatin mass in either domain. The two extremities of this chromosome could also be found on opposite sides of the bud neck, reminiscent of their relative independence during the mitotic cell cycle [46] (Fig 3E).

Thus, chromosome XII assumes a characteristic polarized distribution in arrested cells and at least one other chromosome does not do so. Because the orientation of chromosome XII and the distribution of the nucleolus define a polarity axis for the nucleus, it is of interest to know how this asymmetry is maintained.

### 2. Why does the nucleolus not access the entire nucleus upon arrest?

The nucleolus is the most massive transcriptional unit of the cell and surely includes multiple interacting factors that could cross-link rDNA segments [26, 27, 47]. We therefore propose that the nucleolus is too structured to pass through the bud neck during much of the cell cycle. Indeed, the geometry of the NE (at least in telophase) is known normally to limit translocation between mother and bud domains even for soluble proteins [48].

In the following assays, the cortex of cells expressing a tagged nucleolar protein (Gar1-GFP) was prelabeled with fluorescent ConA. The cells were then arrested and we studied those cells for which the cortex of one domain (the mother) retained the ConA label, while the cortex of the other domain (the bud) was not labeled (Fig 3F). During the arrest interval, the nucleolus remained confined to the mother domain in > 95% of control cells over 4 hours at room temperature.

#### Impact of inhibiting rRNA synthesis on the distribution of the nucleolus

Because the coherence of the nucleolus is known to depend on its transcriptional activity [49, 50], we explored the consequences of inhibiting rRNA synthesis in arrested cells. Inhibition was achieved by using a strain that carries a ts mutation in the large subunit of RNA polymerase I (*rpa190-3*) [51]. In typical protocols (Fig 3G), ConA-labeled cells that express Gar1-GFP were arrested at room temperature and then were reincubated at 23°C or at 36°C for up to 4 hours, all by comparison to controls that were not temperature-sensitive. Cells with large buds were subsequently scored to monitor whether the nucleolar signal remained in the mother domain.

In *rpa190-3 cells* at room temperature, Gar1-GFP persisted in the mother domain; however, upon incubation at 36°C we observed the progressive appearance of Gar1-positive fragments in the bud domain. These included cells in which most signal was still in the mother domain but there was some signal in the bud domain (M > B), and cells in which an approximately equal signal was in both domains (M ~ B). Taken together, ~ 40% of cells had Gar1 fragments in the bud domain within 4 hours (n = 300) (Fig 3G).

In related studies, we attempted to dilate the nucleus at the level of the bud neck by using cells that lack the bud neck kinase, Cla4, that is known to widen the external dimensions of the neck [52]. These cells became prohibitively heterogeneous upon arrest and therefore were not pursued.

The present observations thus support the hypothesis that the massive size of the nucleolus limits its entry into the bud domain.

#### Importance of the nuclear envelope for retention of the nucleolus

A complex of proteins (Csm1, Lrs4) associates with the intergenic spacers of rDNA and governs its recombinational properties. These proteins also associate with a protein of the inner NE (Heh1) [53, 54]. Corresponding deletion strains arrested with good efficiency but deletion of any of these three proteins did not allow Gar1-GFP to access the bud domain of the nucleus in arrested cells over at least 4 hours. Therefore, they are not major players in keeping the nucleolus in the mother domain.

### 3. Septin disorganization causes escape from arrest

Our emphasis to this point has been on events within the nucleus. The septin annulus that surrounds the nucleus could also affect the stability of arrest. We have explored this hypothesis by studying cells that carry ts mutations in septins. To simplify the exposition, we begin by showing that the alternative activator of the APC, Cdh1, can force escape from arrest.

#### Cdh1 can drive cell cycle progression in arrested cells

**Fig 4:**
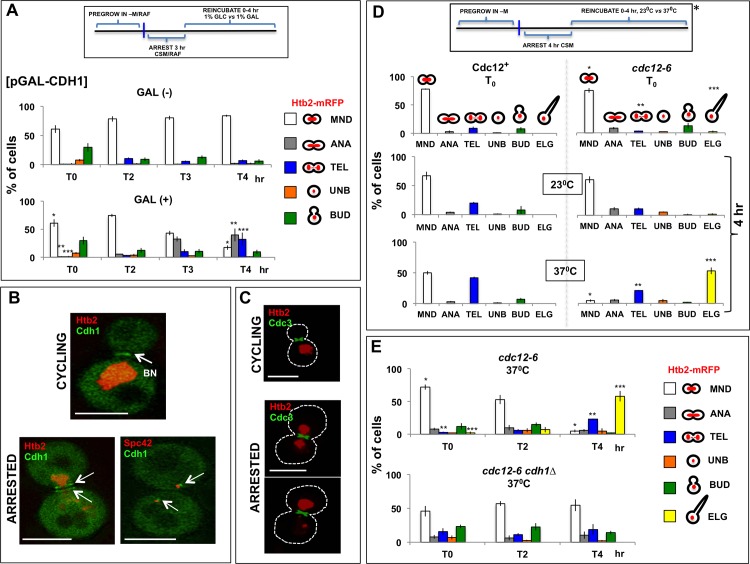
Arrest depends on septin integrity and Cdh1. (A) Induction of Cdh1 expression in arrested cells results in escape. A *MET3-CDC20* strain expressing Htb2-mRFP and carrying a CEN plasmid for induction of Cdh1 expression from a GAL1/10 promoter was arrested for 3 hour in raffinose medium. The cells were then supplemented with 1% galactose (GAL (+)) or 1% glucose (GAL (-)) for 0–4 hours (T0, T2, T3 and T4). By examining the distribution of chromatin, as well as overall shape, the cells were classified into the following categories: MND (chromatin spans the bud neck), ANA (chromatin has begun to stretch), TEL (chromatin has stretched to form two terminal masses that remain connected). In addition, we tabulated unbudded cells (UNB), and cells with small to medium-sized buds (BUD). Note that, induction of Cdh1 expression caused the cell cycle to advance. Statistical significance was evaluated by Student’s *t*-test. In the lower right panel, pairs of bars (before and after galactose induction) bearing the same number of stars have been compared to each other. *P < 0.001, **P < 0.005, and ***P < 0.05. Strain: ATY7545. (B) Localization of Cdh1 in cycling and arrested cells. Cycling cells: In cells expressing Cdh1-GFP and Htb2-mRFP, Cdh1-GFP was detected in the nucleus, the cytoplasm and at the bud side of the bud neck (bud side, arrow). Arrested cells: A *MET3-CDC20* strain expressing Cdh1-GFP and Htb2-mRFP was arrested for 4 hours and examined. Note that Cdh1-GFP localized to the cytoplasm and was found at the bud neck (both sides, even when cytokinesis has not occurred, judging from the distribution of Htb2-mRFP) and adjacent to SPBs (arrow). In the bottom image, the Spc42-CFP signal was pseudocolored in red. Strains: ATY7652 and ATY7710. (C) Septin distribution in arrested cells. A *MET3-CDC20* strain expressing Htb2-mRFP and a tagged septin, GFP-Cdc3, was arrested for 4 hours and imaged. Note that the septin hourglass encircled the bud neck and that its organization did not obviously change during transits. Strain: ATY3847. (D) Destabilization of arrest in a septin mutant. A *MET3-CDC20* strain expressing Htb2-mRFP (left panels) and an equivalent strain that carried the *cdc12-6* ts septin mutation (right panels) were arrested at 23°C for 4 hours. The cells were then further incubated at 23°C or at 37°C for 0, 2, or 4 hours and examined. They were assigned to categories as above. An additional category (ELG) is for single cells with an elongated bud. Note that the cell cycle advanced in the ts mutant strain to the point that almost no MND cells remained, with parallel accumulation of cells with elongated buds. Arrest remained stable in the Ts+ cells. Statistical significance was evaluated by Student’s *t*-test. In the top right and bottom right panels, pairs of bars (i.e. before septin inactivation *vs* after inactivation) bearing the same number of stars have been compared to each other. *P < 0.0001, **P < 0.0005, and ***P < 0.0005. Strains: ATY4336 and ATY3249. (E) Cdh1 is required for efficient escape. A *MET3-CDC20 cdc12-6* strain expressing Htb2-mRFP and an equivalent strain lacking *CDH1* were arrested and then challenged by shifting to 37°C for 0–4 hours, essentially as in Fig 4D. Note that the cell cycle advanced only if *CDH1* was present. Statistical significance was evaluated by Student’s *t*-test. In the upper panel, pairs of bars (i.e. before and after septin inactivation) bearing the same number of stars have been compared to each other. *P < 0.0001, **P < 0.0005, and ***P < 0.0005. Strains: ATY4336 and ATY7534.

Depletion of Cdc20 effectively interrupts cell cycle progression. Considering that the second activator of the APC, Cdh1, normally stimulates the APC at the very end of the cell cycle [13, 55–60], could it also affect the stability of arrest? In fact, we observed that induction of Cdh1 in arrested cells did cause completion of the cell cycle, as judged from the distribution of Htb2-mRFP (Fig 4A). Related observations of cell cycle progression beyond a block imposed by nocodazole have been made in cells that lack Bub2 and therefore cannot activate the spindle position checkpoint. Here too, Cdh1 is required for progression. Parallel investigation of Bub2+ cells also show that induction of Cdh1 can promote progression in the presence of nocodazole [61].

As previously reported, we found that Cdh1-GFP localized to the nucleus during most of the normal cell cycle and then distributed throughout the cytoplasm in medium- to large-budded cells (n = 80). In ~ 6% of these latter cells, the GFP signal formed a single ring adjacent to the bud neck, as in earlier studies [55]. In arrested cells, Cdh1-GFP was found throughout the cytoplasm, and ~ 15% of these cells had Cdh1-GFP rings on both sides of the bud neck. ~ 28% of cells also localized Cdh1-GFP adjacent to SPBs (n = 80) (Fig 4B).

#### Septin disruption allows cell cycle progression if Cdh1 is present

The tagged septin, GFP-Cdc3, can be detected at the bud neck of all arrested cells (Fig 4C). To learn whether septins contribute to arrest, we arrested cells that express Htb2-mRFP and carry the ts septin mutation, *cdc12-6*. After 4 hours, the cells were reincubated at 23°C *vs* 37°C for up to 4 hours and we then quantitated several categories of cells: (i) Cells with chromatin extending between mother and bud (MND), (ii) Cells showing symmetric anaphase-like stretching of chromatin between the mother and bud, (iii) Telophase-like cells with two fully-separated chromatin masses without cytokinesis, and (iv) Cells that had accomplished cytokinesis, some of which had an elongated bud that tapered toward its distal end (as in *cdc12-6* haploid cells incubated at the restrictive temperature). After 4 hours at 23°C, arrest was stable, whereas at 37°C there was a major reduction in the number of arrested cells and a corresponding increase in cells that had accomplished cytokinesis (Fig 4D).

In equivalent experiments with Ts+ wildtype cells that express Htb2-mRFP, we observed that there was minimal completion of the cell cycle, although, interestingly, there was some further separation of chromatin at 37°C. If this is caused by limited activation of the APC [62], it is surprising that cells do not progress further. To evaluate the generality of the observations made with *cdc12-6*, we also studied cells that carry a different septin ts mutation (*cdc11-6*) and we observed equivalent escape after arrest. In arrested *cdc12-6* cells that expressed the tagged septin, GFP-Cdc3, we observed, as expected, a corresponding disruption of organization of this septin upon incubation at 37°C (S4 Fig).

Moreover, Cdh1 is required for escape: When we deleted *CDH1* from *cdc12-6* cells, arrested these cells for 4 hours, and then shifted them to 37°C, we did not detect cell cycle progression over the following 4 hours at 37°C (n > 500) (Fig 4E). Full disassembly of the actomyosin ring at the end of the cell cycle requires Cdh1 [60]. One model to explain the septin requirement for arrest therefore postulates that the intact septin hourglass structure normally inhibits Cdh1 and that perturbation of the hourglass—as in *cdc12-6* –relieves this inhibition, thereby allowing cell cycle progression.

Because septin perturbation (e.g. in *cdc12-6*) disorganizes actin filaments [63, 64], we inquired whether arrest is linked to integrity of actin distribution. In cycling cells, we observed that actin filaments labeled with a GFP-tagged actin-binding protein (Abp140) traversed the cytoplasm of both mother and bud and that labeled patches were strongly concentrated at the bud cortex [8, 65, 66]. In arrested cells, we found that Abp140-3GFP was nearly symmetrically distributed. In both domains, it highlighted filaments and patches at the cortex as well as filaments that traversed the cytoplasm (S5 Fig). Moreover, as in cycling cells, the tagged formin, 3GFP-Bnr1, formed a ring at the bud neck in arrested cells (S5 Fig) [63, 64]. This seemingly normal distribution of actin is however not required for transits. Thus, once cells had been arrested, the addition of latrunculin A at doses that have a profound and rapid effect on filamentous actin (15 μM) did not affect transits over 20 minutes.

The near-symmetry of the mother and bud is also evident in the distribution of vacuoles that contact the surface of the nucleus in both domains in arrested cells [67] (S6 Fig).

In sum, induction of Cdh1 in arrested cells is sufficient to drive completion of the cell cycle. Moreover, Cdh1 (and by implication the APC) are required in order for septin inactivation to allow cell cycle progression.

### 4. Distribution of cell cycle regulatory proteins upon arrest

Upon spindle elongation during the normal cell cycle, one SPB enters the bud. The Tem1 GTPase, that is at the top of the mitotic exit signaling cascade, localizes to this bud and is activated upon entry by its GAP, Bfa1/Bub2. Activation can occur since Tem1 and Bfa1/Bub2 in the bud domain are spatially separate from the inhibitory Kin4 kinase that localizes primarily to the mother cortex and to the mother SPB [68, 69]. Moreover, upon entry into the bud, Lte1 in the bud opposes the action of any Kin4 that may be present [70–72].

To better understand the stability of arrest, we localized proteins that control mitotic exit. As indicated in S7A Fig, in arrested cells Tem1-GFP and Bfa1-GFP were present on both SPBs. Stability of the arrest thus could rely on the brevity of residence of each Tem1-positive SPB in the bud domain. We also observed that Lte1-GFP remained restricted to the bud domain and that the inhibitory kinase, Kin4-GFP, was at the cortex of both mother and bud. The relocation of Kin4 could contribute to arrest; however, cells in which *KIN4* (or *LTE1*) was deleted did arrest with good efficiency when these strains were equipped with the *MET3-CDC20* cassette, allowing depletion of Cdc20: As for wildtype cells, > 80% arrested over 4 hr, judging from accumulation of large-budded cells and the distribution of chromatin in replicate experiments.

S7B Fig also shows that Lte1-GFP relocated to both mother and bud domains of arrested cells upon inactivation of *cdc12-6*, even when the chromatin still spanned the bud neck. Similar observations have previously been reported for septin mutants in the absence of cell cycle arrest [74, 75].

S7C Fig summarizes these distributions by comparison to the normal cycle and to cells arrested at the spindle position checkpoint [73].

## Discussion

Orderly progression through the cell cycle depends on checkpoints that impose arrest if critical features of the cell are not prepared to advance. Without these surveillance mechanisms, catastrophic mistakes occur [66, 70, 73, 76–78]. From the point of view of nuclear dynamics, we find that the arrest response to DNA damage (elicited by exposing single-stranded DNA in *cdc13-1* [79, 80]) is very similar to the Cdc20 arrest (S8 Fig).

Upon Cdc20 depletion, cells are in a metaphase-like state that is largely symmetrical. Thus, the size of the bud is nearly equal to that of the mother and the vacuoles and the actin cytoskeleton assume a similar distribution in both domains. Nevertheless, the cortical polarity determinant, Lte1 remains restricted to the bud domain. Additionally, the nucleus retains its polarity, in that the nucleolus and the long arm of chromosome XII remain in the mother domain. In this condition, cytoplasmic microtubules pull at the SPBs, causing dramatic displacement of the chromatin mass. The dynamics are such that individual SPBs visit both mother and bud domains with a surprising rapidity, considering their integration into the NE. The apparent fragmentation of the centromere and kinetochore clusters that we have observed is presumably due to these dynamic forces.

Arrest is associated with at least two potentially stabilizing relations for the NE: intranuclear events that limit displacement of the nucleolus, and possible association of the external face of the NE with septins—that could oppose displacement of the nucleus from the bud neck.

### Events within the nucleus of arrested cells

The nucleolus and long arm of chromosome XII show distinctive behavior during the normal cell cycle, in that they lag behind the chromatin mass during anaphase. Telomere XIIR might have a specific link to the inner aspect of the NE in the mother domain. Nevertheless, it is not obvious that telomere XIIR is structurally different from other telomeres [81]. We therefore favor the seemingly mechanical explanation for this lag, i.e. that the mass of the nucleolus is less malleable than the rest of chromatin. This restriction could correspondingly delay normal cell cycle progression.

The nucleolar lag in wildtype cells has previously been attributed to the delay of release of Cdc14 phosphatase from the nucleolus, which is prompted after metaphase, first by the FEAR network and then by the mitotic exit network [82, 83]. Thus, activation of Cdc14 as well as cohesin cleavage seem not to be needed to cause passage of the nucleolus across the neck [69, 83].

Consistent with the suggestion that the size of the nucleolus impedes its entry into the bud, in cells that have only a single copy of rDNA (transcribed by RNA polymerase 2) and therefore lack a classical structured nucleolus, there is little or no lag of rDNA segregation during the cell cycle. Moreover, mutation of Cdc14 scarcely affects the timing of nucleolar segregation in such cells [83, 84].

### Events outside the nucleus in arrested cells

The biochemistry of the bud neck, including its multiple kinases, has been investigated in great detail [14, 85, 86]; however, there is little information concerning the physical relation between the outer aspect of the NE and the cell cortex at this level. Septin integrity is required, however, to limit mother-bud diffusion of proteins of the inner aspect of the cell cortex, ER membrane proteins, and membrane proteins of the outer membrane of the NE [17–19]. A septin annulus that surrounds the midzone of yeast zygotes is also critical for restricting precocious fusion between parental mitochondria [87]. Interestingly, mechanical stress exerted on the NE elicits an ATR-dependent checkpoint in animal cells [88].

The importance of septins for arrest could be structural since the septin ring is appropriately situated to contact both the cell cortex and the NE. Other possible explanations of the importance of septins could involve their ability to sequester critical factors that normally enter into the nucleus and regulate cell cycle progression [89]. A further possible contribution to the destabilization of arrest could be that the intact septin hourglass inhibits Cdh1 at the bud neck. The mechanism by which this could be achieved remains to be determined; however, it could also explain why Cdh1 normally is activated at the very end of the cell cycle, i.e. when the septin hourglass becomes reorganized to form two parallel rings, and then disassembles.

### The importance of the bud neck during arrest

Our studies show that the transcriptional activity of the nucleolus as well as septin integrity regulate features of cell cycle arrest. Lesions that impair these controls could contribute to untimely cell cycle progression, with inappropriate consequences. Because the supramolecular complexes that participate in these events require the precise contributions of many proteins, arrest could be impaired for any cells in which single components malfunction.

## Supporting information

S1 Fig Fragmentation of centromere and kinetochore clusters. SPB mutant cells (*spc42-10*) expressing Cse4-GFP or Ndc10-GFP were shifted to 37°C for increasing periods of time and examined. Note especially the examples of cells that have more than 1–2 foci and can even appear pulverized. Quantitation of these data is given in the bar graphs. Strains: ATY7203 and ATY7204.
(TIFF)

S2 Fig The nucleolus remains in the mother domain. A small-budded *MET3-CDC20* cell expressing Htb2-mRFP and Gar1-GFP was grown in methionine-free medium, transferred to methionine-containing medium at t = 0, and imaged at 23°C. Note that chromatin entered the bud after 2 hours and that the nucleolar signal remained in the mother. By the final time point the size of the bud and mother were comparable. Strain: ATY3175.
(TIFF)

S3 Fig Rap1 localization. A *MET3-CDC20* strain expressing GFP-Rap1 and Htb2-mRFP was examined before and after arrest for 4 hours. Note that, as in cycling cells, the GFP signal formed multiple foci. They were, however, less frequently concentrated at the edge of chromatin than in cycling cells. Strains: ATY6461.
(TIFF)

S4 Fig Disruption of septin organization in arrested *cdc12-6* cells. A *MET3-CDC20 cdc12-6* strain expressing Htb2-mRFP and GFP-Cdc3 was arrested at the permissive temperature (T0). The cells were then further incubated at 23°C or at 37°C (restrictive temperature) for 2 or 4 hours and examined. Note that the septin hourglass remained intact at the permissive temperature (upper two panels) but became disorganized at the restrictive temperature (lower panels). Further incubation at 37°C led to cytokinesis. Such disorganization was not seen when wildtype strains were incubated at 37°C. Strain ATY8270.
(TIFF)

S5 Fig Distribution of Abp140 and the formin, Bnr1. A *MET3-CDC20* strain that expressed tagged forms of the actin-binding protein, Abp140, or the formin, Bnr1, was arrested for 4 hours and examined. Note that GFP3-Abp140 was symmetrically distributed, forming filaments in both domains and patches at the cortex of both domains. As in cycling cells, Bni1-GFP formed a septin-like annulus around the bud neck. Strains: ATY7604 and ATY7615.
(TIFF)

S6 Fig Vacuoles are distributed symmetrically. A *MET3-CDC20* strain expressing Htb2-mRFP was arrested for 4 hours and then stained with FM4-64. Note that vacuoles were found apposed to the nucleus in both domains. Strain: ATY3249.
(TIFF)

S7 Fig Distribution of cell cycle regulatory proteins upon cell cycle arrest. **(A)** Live cell localization of cell cycle regulatory proteins. Both cycling cells and cells that had been arrested for 4 hours expressed the indicated GFP-tagged proteins were examined. The following characteristics were observed:
Tem1-GFP localized to both SPBs upon arrest.The behavior of Bfa1-GFP (and Bub2-GFP) was similar to that of Tem1.The distribution of Lte1-GFP did not change upon arrest.Kin4-GFP shifted from its location at the mother cortex and mSPB to being present also at the bud cortex upon arrest.
Strains: ATY6081, ATY6083, ATY6085, ATY6095 and ATY6835.
**(B)** Distribution of Lte1-GFP in arrested *cdc12-6* cells. A *MET3-CDC20 cdc12-6* strain expressing Htb2-mRFP and Lte1-GFP was arrested for 4 hours at 23°C and then shifted to 37°C. The images were acquired before and after 4 hours at 37°C. Note that Lte1-GFP remains at the cortex of the bud. Strain: ATY7611.
**(C)** Model summarizing the distribution of cell cycle regulatory proteins upon Cdc20 depletion by comparison to the spindle position checkpoint (SPOC) arrest and to cycling cells. The distributions observed upon Cdc20 depletion are likely to resemble those that characterize activation of the spindle assembly checkpoint *per se*, and is therefore labeled as such. The spindle and SPBs are represented by the black lines that end in circles. As shown for normal metaphase, the green stripes are in the bud domain that normally includes Lte1 and therefore is considered to activate cell cycle progression. The red stripes correspondingly are in the mother domain, that is considered inhibitory. Upon arrest (middle), Lte1 remains in the bud domain, while Kin4 is in both domains. The spindle shifts between domains. When Cdc12 is inactivated (*cdc12-6*, 37°C, 4 hours), both Lte1 and Kin4 become uniformly distributed and the cell cycle continues. Judging from the literature [73], at the spindle position checkpoint (SPOC), Lte1 and Kin4 remain in single domains.
(TIFF)

S8 Fig Nuclear dynamics upon DNA damage arrest. The DNA damage checkpoint monitors the presence of single-stranded DNA and related DNA lesions. This response can be triggered by inactivating Cdc13, that normally binds telomeric single-stranded DNA. For this purpose, it is sufficient to incubate *cdc13-1* strains at 30°C.
As shown, after incubation at 30°C, chromatin exhibited transits between both lobes of the nucleus, the spindle transected the chromatin, and the nucleolus remained in the maternal lobe. The two time-lapse series illustrate cells that had been arrested for 3 hours at 30°C. The upper series shows the distribution of chromatin (Htb2-mRFP) and the nucleolus (Gar1-GFP). Arrows indicate the direction in which chromatin was about to move. The lower series illustrates the distribution of the NE, chromatin and the spindle (Nup49-GFP, Htb2-mRFP and Tub1-GFP). Strains: ATY3424 (upper panel) and ATY3543 (lower panels).
(TIFF)

S1 Table Strain List. (DOCX)

S2 Table Plasmid List. (DOCX)

S3 Table Table for Fig 2F. (PPTX)

S4 Table Table for Fig 3G. (PPTX)

S5 Table Table for Fig 4A. (PPTX)

S6 Table Table for Fig 4D. (PPTX)

S7 Table Table for Fig 4E. (PPTX)

S8 Table Table for S1 Fig. (PPTX)

## Abbreviations

APC: anaphase promoting complex
MND: medial nuclear division
NE: nuclear envelope
SAC: spindle assembly checkpoint (also known as the spindle checkpoint or mitotic checkpoint)
SPB: spindle pole body
SPOC: spindle position checkpoint
