# Supplementary figures and images for: Nucleolar asymmetry and the importance of septin integrity upon cell cycle arrest
Source: PLoS One. 2017 Mar 24;12(3):e0174306. doi: 10.1371/journal.pone.0174306 (PMC5365125; doi:10.1371/journal.pone.0174306)

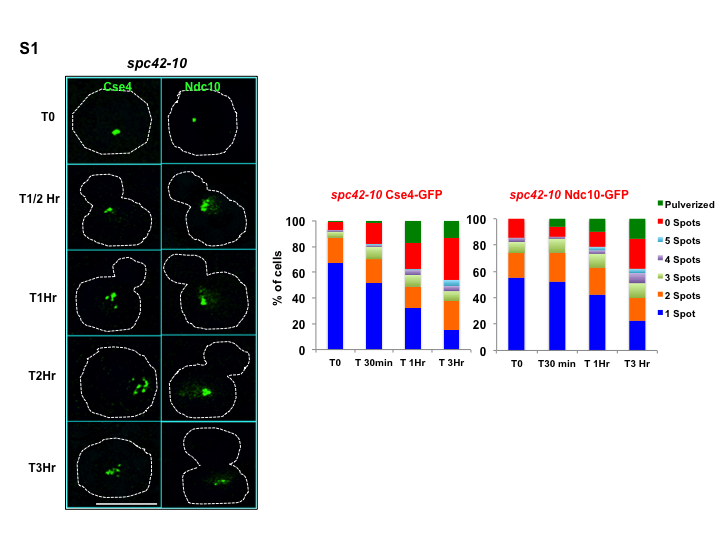

Supplement: S1 Fig — SPB mutant cells (spc42-10) expressing Cse4-GFP or Ndc10-GFP were shifted to 37°C for increasing periods of time and examined. Note especially the examples of cells that have more than 1–2 foci and can even appear pulverized. Quantitation of these data is given in the bar graphs. Strains: ATY7203 and ATY7204. (TIFF) [file pone.0174306.s001.tiff]

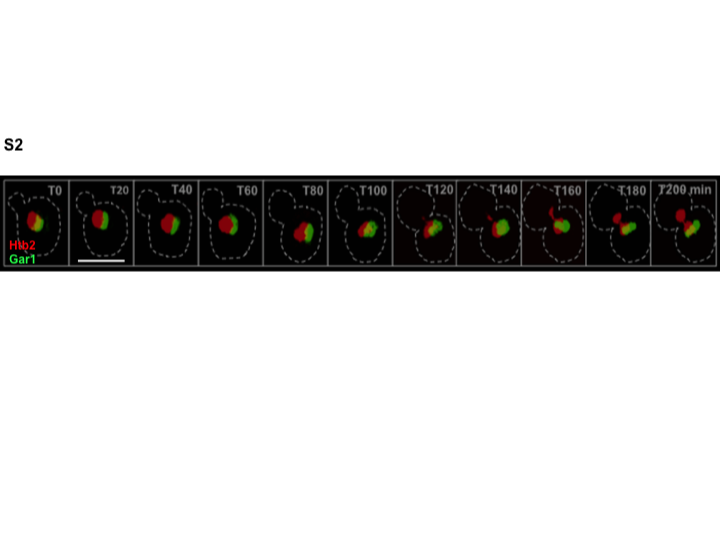

Supplement: S2 Fig — A small-budded MET3-CDC20 cell expressing Htb2-mRFP and Gar1-GFP was grown in methionine-free medium, transferred to methionine-containing medium at t = 0, and imaged at 23°C. Note that chromatin entered the bud after 2 hours and that the nucleolar signal remained in the mother. By the final time point the size of the bud and mother were comparable. Strain: ATY3175. (TIFF) [file pone.0174306.s002.tiff]

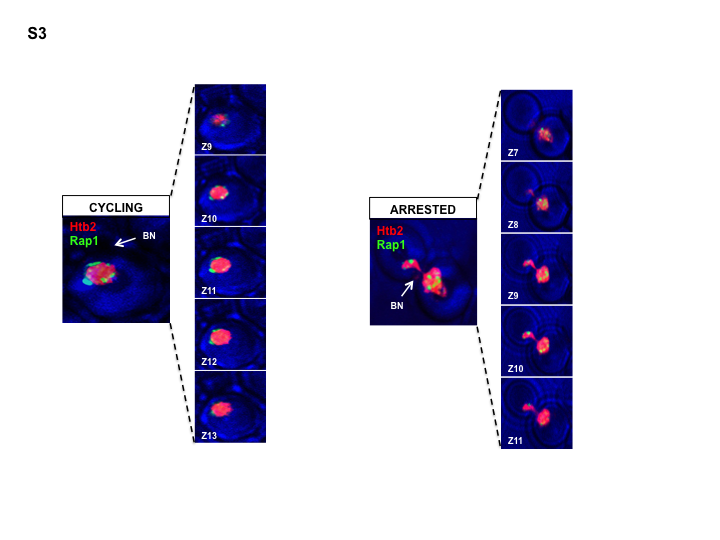

Supplement: S3 Fig — A MET3-CDC20 strain expressing GFP-Rap1 and Htb2-mRFP was examined before and after arrest for 4 hours. Note that, as in cycling cells, the GFP signal formed multiple foci. They were, however, less frequently concentrated at the edge of chromatin than in cycling cells. Strains: ATY6461. (TIFF) [file pone.0174306.s003.tiff]

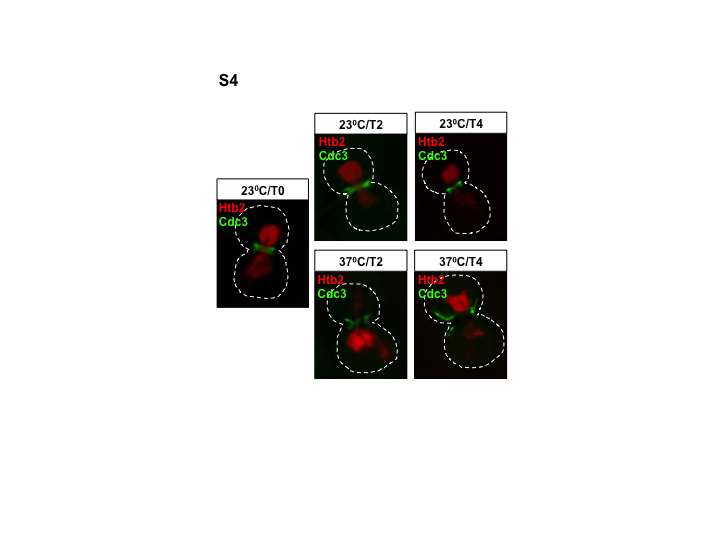

Supplement: S4 Fig — A MET3-CDC20 cdc12-6 strain expressing Htb2-mRFP and GFP-Cdc3 was arrested at the permissive temperature (T0). The cells were then further incubated at 23°C or at 37°C (restrictive temperature) for 2 or 4 hours and examined. Note that the septin hourglass remained intact at the permissive temperature (upper two panels) but became disorganized at the restrictive temperature (lower panels). Further incubation at 37°C led to cytokinesis. Such disorganization was not seen when wildtype strains were incubated at 37°C. Strain ATY8270. (TIFF) [file pone.0174306.s004.tiff]

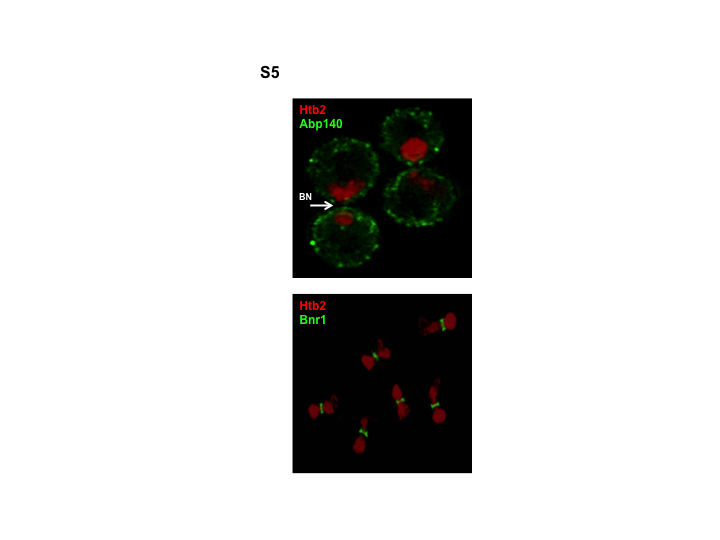

Supplement: S5 Fig — A MET3-CDC20 strain that expressed tagged forms of the actin-binding protein, Abp140, or the formin, Bnr1, was arrested for 4 hours and examined. Note that GFP3-Abp140 was symmetrically distributed, forming filaments in both domains and patches at the cortex of both domains. As in cycling cells, Bni1-GFP formed a septin-like annulus around the bud neck. Strains: ATY7604 and ATY7615. (TIFF) [file pone.0174306.s005.tiff]

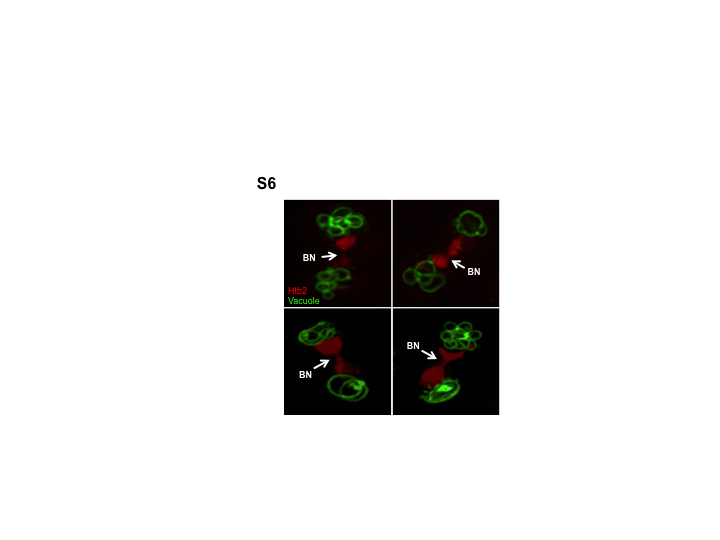

Supplement: S6 Fig — A MET3-CDC20 strain expressing Htb2-mRFP was arrested for 4 hours and then stained with FM4-64. Note that vacuoles were found apposed to the nucleus in both domains. Strain: ATY3249. (TIFF) [file pone.0174306.s006.tiff]

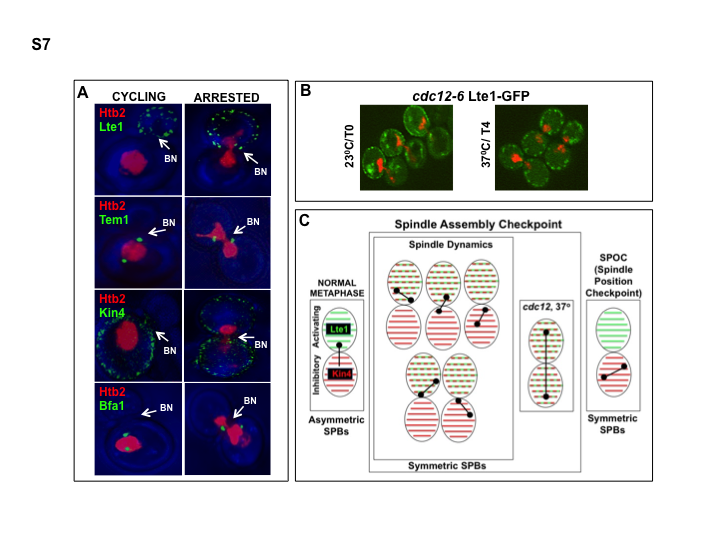

Supplement: S7 Fig — (A) Live cell localization of cell cycle regulatory proteins. Both cycling cells and cells that had been arrested for 4 hours expressed the indicated GFP-tagged proteins were examined. The following characteristics were observed: Tem1-GFP localized to both SPBs upon arrest.The behavior of Bfa1-GFP (and Bub2-GFP) was similar to that of Tem1.The distribution of Lte1-GFP did not change upon arrest.Kin4-GFP shifted from its location at the mother cortex and mSPB to being present also at the bud cortex upon arrest. Strains: ATY6081, ATY6083, ATY6085, ATY6095 and ATY6835. (B) Distribution of Lte1-GFP in arrested cdc12-6 cells. A MET3-CDC20 cdc12-6 strain expressing Htb2-mRFP and Lte1-GFP was arrested for 4 hours at 23°C and then shifted to 37°C. The images were acquired before and after 4 hours at 37°C. Note that Lte1-GFP remains at the cortex of the bud. Strain: ATY7611. (C) Model summarizing the distribution of cell cycle regulatory proteins upon Cdc20 depletion by comparison to the spindle position checkpoint (SPOC) arrest and to cycling cells. The distributions observed upon Cdc20 depletion are likely to resemble those that characterize activation of the spindle assembly checkpoint per se, and is therefore labeled as such. The spindle and SPBs are represented by the black lines that end in circles. As shown for normal metaphase, the green stripes are in the bud domain that normally includes Lte1 and therefore is considered to activate cell cycle progression. The red stripes correspondingly are in the mother domain, that is considered inhibitory. Upon arrest (middle), Lte1 remains in the bud domain, while Kin4 is in both domains. The spindle shifts between domains. When Cdc12 is inactivated (cdc12-6, 37°C, 4 hours), both Lte1 and Kin4 become uniformly distributed and the cell cycle continues. Judging from the literature [73], at the spindle position checkpoint (SPOC), Lte1 and Kin4 remain in single domains. (TIFF) [file pone.0174306.s007.tiff]

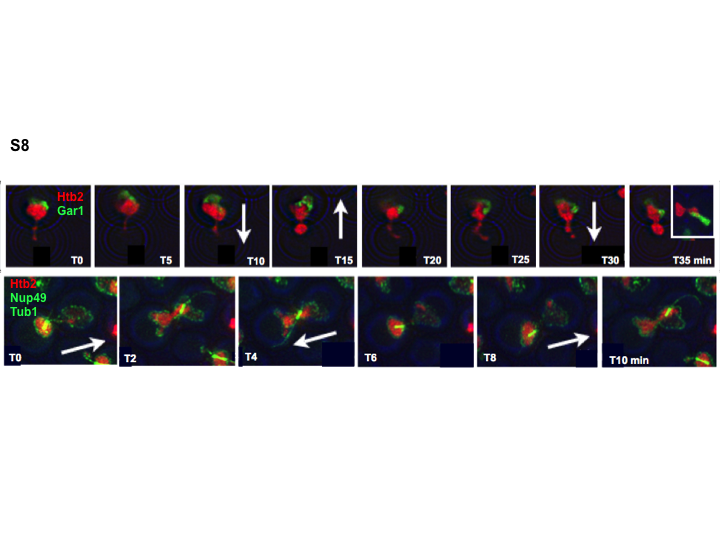

Supplement: S8 Fig — The DNA damage checkpoint monitors the presence of single-stranded DNA and related DNA lesions. This response can be triggered by inactivating Cdc13, that normally binds telomeric single-stranded DNA. For this purpose, it is sufficient to incubate cdc13-1 strains at 30°C. As shown, after incubation at 30°C, chromatin exhibited transits between both lobes of the nucleus, the spindle transected the chromatin, and the nucleolus remained in the maternal lobe. The two time-lapse series illustrate cells that had been arrested for 3 hours at 30°C. The upper series shows the distribution of chromatin (Htb2-mRFP) and the nucleolus (Gar1-GFP). Arrows indicate the direction in which chromatin was about to move. The lower series illustrates the distribution of the NE, chromatin and the spindle (Nup49-GFP, Htb2-mRFP and Tub1-GFP). Strains: ATY3424 (upper panel) and ATY3543 (lower panels). (TIFF) [file pone.0174306.s008.tiff]
