# Supplementary material for: Nucleolar asymmetry and the importance of septin integrity upon cell cycle arrest
Source: PLoS One. 2017 Mar 24;12(3):e0174306. doi: 10.1371/journal.pone.0174306 (PMC5365125; doi:10.1371/journal.pone.0174306)
Supplement: S1 Table — (DOCX) [file pone.0174306.s009.docx]

| **S1 Table. Strain List*** | | | |
| --- | --- | --- | --- |
| **Strain** | **Relevant Genotype** | **Reference** | **Plasmid** |
| ATY1803 | MAT a *cdc13-1* | 4529-131, Koshland, D |  |
| ATY2089 | MAT a *cdc12-6* | #743, Bi, E |  |
| ATY2201 | MAT a *MET3-HA3-CDC20*::*TRP1* | K9022, Nasmyth, K |  |
| ATY2289 | MAT α HTB2-mRFP::G418 | This study |  |
| ATY2416 | MAT a HTB2-mRFP [Gar1-GFP] | This study | pAT624 |
| ATY2662 | MAT a TelVL-YFP-TetO TelVR-CFP-LacO | GA2199, Gasser, S |  |
| ATY3175 | ATY3249 [Gar1-GFP] | This study | pAT624 |
| ATY3215 | MAT a *cdc13-1* HTB2-mRFP::G418 | This study |  |
| ATY3249 | MAT a *MET3-HA3-CDC20*::*TRP1* HTB2-mRFP::G418 | This study |  |
| ATY3369 | MAT a *cdc13-1* HTB2-mRFP Scc1-HA *GAL-ESP1*-GFP | This study | pAT1002 |
| ATY3375 | MAT a ATY3249 Scc1-HA *GAL-Esp1*-GFP | This study | pAT1002 |
| ATY3424 | ATY3215 [Gar1-GFP] | This study | pAT624 |
| ATY3539 | ATY3249 [Tub1-GFP] | This study | pAT1011 |
| ATY3543 | ATY3215 [Tub1-GFP] [Nup49-GFP] | This study | pAT994 pAT635 |
| ATY3847 | ATY3249 [GFP-Cdc3] | This study | pAT1125 |
| ATY4336 | ATY3539 *cdc12-6* | This study; 743, Bi, E |  |
| ATY4435 | ATY3249 [Nup49-GFP] | This study | pAT986 |
| ATY6081 | ATY3249 Lte1-GFP | This study |  |
| ATY6083 | ATY3249 Tem1-GFP | This study |  |
| ATY6085 | ATY3249 Kin4-GFP | This study |  |
| ATY6095 | ATY3249 Bfa1-GFP | This study |  |
| ATY6196 | ATY3249 Cse4-GFP | This study |  |
| ATY6461 | ATY3249 [GFP-Rap1] | This study | pAT1432 |
| ATY6585 | ATY3249 Spc42-mRFP [H2A-GFP] | This study | pAT947 |
| ATY6790 | rDNA-YFP-TetO:487-ChrXII Net1-CFP – locus D | CCG1328, Aragon, L |  |
| ATY6791 | rDNA-YFP-TetO:1061-ChrXII (TelR) Net1-CFP – locus F | CCG1329, Aragon, L |  |
| ATY6833 | ATY3249 Cse4-GFP^INT-Tagged^ | This study; MBY507, Wisniewski, J |  |
| ATY6835 | ATY3249 Bub2-GFP | This study |  |
| ATY6850 | MAT a *kin4*∆ | ESM2263, Pereira, G |  |
| ATY6863 | ATY3249 [Cdc14-GFP] | This study |  |
| ATY6877 | ATY3249 *cdc11-6* | This study; 742, Bi, E |  |
| ATY6882 | ATY3249 [Ndc10-GFP] | This study | pAT1172 |
| ATY7009 | ATY3249 *kin4*∆ | This study |  |
| ATY7063 | MAT a Spc42-CFP | From JK1659, Kilmartin, J. |  |
| ATY7102 | rDNA-YFP-TetO:1061-ChrXII Net1-CFP – locus F | From CCG1329, Aragon, L |  |
| ATY7135 | ATY3539 Spc42-CFP | This study |  |
| ATY7203 | MAT a *spc42-10* [Ndc10-GFP] | This study | pAT1172 |
| ATY7204 | MAT a *spc42-10* [Cse4-GFP] | This study | pAT1171 |
| ATY7241 | rDNA-YFP-TetO:487-ChrXII Net1-CFP – locus D | From CCG1328, Aragon, L. |  |
| ATY7272 | ATY3175 TelVL-YFP-TetO TelVR-CFP-LacO | This study |  |
| ATY7473 | MAT a/MAT α Brn1-GFP Sik1-mRFP | This study, Huh, W. |  |
| ATY7534 | MAT α ATY4336 *cdh1*∆ | This study; AA1110, Amon, A |  |
| ATY7545 | ATY3249 [*GAL-CDH1*] | This study | pAT1518 |
| ATY7604 | ATY3249 [Abp140-GFP] | This study | pAT1381 |
| ATY7611 | ATY3249 Lte1-GFP *cdc12-6* | This study |  |
| ATY7615 | ATY3249 [Bni1-GFP] | This study | pAT1380 |
| ATY7633 | MAT a *MET3-CDC20* *GAL-ESP1* TelLXII (lacO tag at MMP1) – locus A | 1780, Uhlmann, F. |  |
| ATY7634 | MAT a *MET3-CDC20* *GAL-ESP1* CENXII (lacO tag at YLR003c-1) – locus B | 1781, Uhlmann, F. |  |
| ATY7637 | MAT a *MET3-CDC20* *GAL-ESP1* 290kb from TelXIIR (lacO tag at REC102) – locus E | 1784, Uhlmann, F. |  |
| ATY7652 | ATY3249 [Cdh1-GFP] | This study | pAT1408 |
| ATY7681 | MAT a *lrs4Δ* *MET3-CDC20* [Gar1-GFP] | This study | pAT624 |
| ATY7684 | MAT a *csm1Δ* *MET3-CDC20* [Gar1-GFP] | This study | pAT624 |
| ATY7710 | ATY3249 Spc42-CFP [Cdh1-GFP] | This study | pAT1408 |
| ATY7724 | MAT a *heh1Δ* *MET3-CDC20* [Gar1-GFP] | This study | pAT624 |
| ATY7733 | MAT a *MET3-CDC20* TetO tag195kb from TelXIIL – locus C | From CCG1326, Aragon, L. | pAT624 |
| ATY7760 | ATY3249 *rpa190-3* [Gar1-GFP] | From Nomura, M. 401 | pAT624 |
| ATY8197 | MAT a *cdc12-6 MET3-CDC20* Htb2-mRFP [Nup49-GFP] | This study | pAT986 |
| ATY8270 | MAT a *cdc12-6* *MET3-CDC20* Htb2-mRFP [GFP-Cdc3] | This study | pAT1125 |
| * All strains with the designation, ATY3249 share the following characteristics: MAT a *MET3-HA3-CDC20*::*TRP1* HTB2-mRFP::G418 | | | |
