# Supplementary material for: Nucleolar asymmetry and the importance of septin integrity upon cell cycle arrest
Source: PLoS One. 2017 Mar 24;12(3):e0174306. doi: 10.1371/journal.pone.0174306 (PMC5365125; doi:10.1371/journal.pone.0174306)
Supplement: S2 Table — (DOCX) [file pone.0174306.s010.docx]

| **S2 Table. Plasmid List** | | |
| --- | --- | --- |
| **Plasmid** | **Description** | **Reference** |
| pAT624 | Gar1-GFP (CEN/URA3) | pZUT3, Gas, N |
| pAT635 | Nup49-GFP (CEN/LEU2) | pUN100, Doye, V |
| pAT947 | H2A-(GFP)_2_ (CEN/URA3) | pNM22 Pemberton, L |
| pAT986 | GFP-Nup49 (CEN/URA3) | #251, Doye, V |
| pAT994 | Tub1-GFP (YIp/LEU2) | pBJ1351, Cooper, J |
| pAT1002 | GAL-Esp1-GFP (YIp/URA3) | Reed, S |
| pAT1011 | Tub1-GFP (YIp/URA3) | pBJ1333, Cooper, J |
| pAT1125 | GFP-Cdc3 (CEN/URA3) | Bi, E. |
| pAT1171 | Cse4-GFP (YIp/TRP1) | pRB759, Baker, R |
| pAT1172 | Ndc10-GFP (YIp/TRP1) | pRB758, Baker, R |
| pAT1381 | Abp140-3GFP (YIp/LEU2) | pB1994, Pellman, D |
| pAT1384 | 3GFP-Bnr1 (CEN/URA3) | pB2617, Pellman, D |
| pAT1408 | Cdh1-GFP (CEN/URA3) | MJ962, Peter, M |
| pAT1432 | GFP-Rap1 (CEN/URA3) | Donaldson, A |
| pAT1476 | Spc42-TFP (CEN/URA3) | Frydman, J |
| pAT1518 | GAL-Cdh1 (CEN/URA3) | Boone, C |
| pAT1519 | MET3-HA-Cdc20 (YIp/TRP1) | p109, Uhlmann, F |
| pAT1520 | MET3-HA-Cdc20 (YIp/LEU2) | p640, Uhlmann, F |
