# Supplementary material for: Nucleolar asymmetry and the importance of septin integrity upon cell cycle arrest
Source: PLoS One. 2017 Mar 24;12(3):e0174306. doi: 10.1371/journal.pone.0174306 (PMC5365125; doi:10.1371/journal.pone.0174306)
Supplement: S3 Table — (PPTX) [file pone.0174306.s011.pptx]

## Slide 1
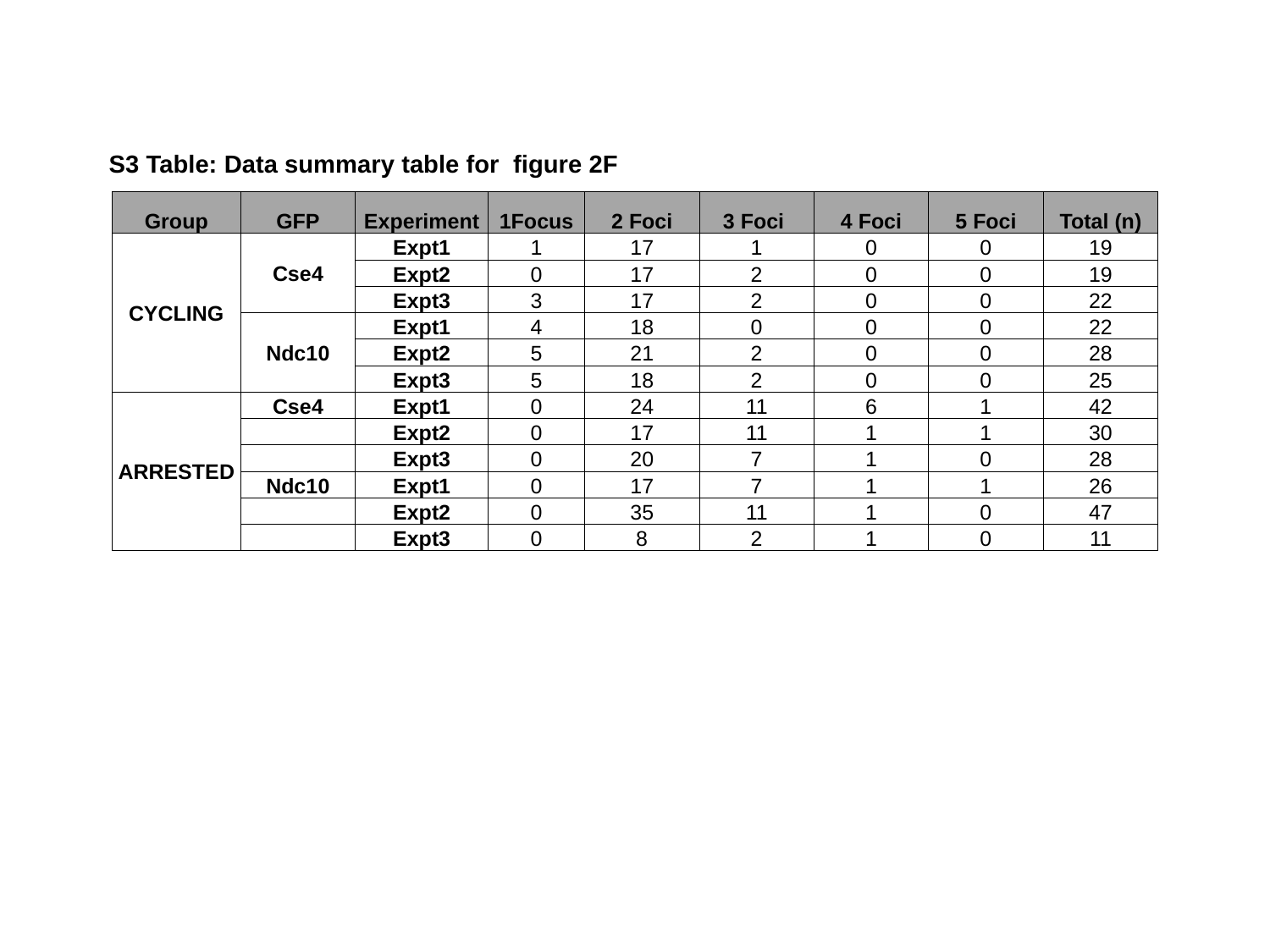

S3 Table: Data summary table for figure 2F
| Group | GFP | Experiment | 1Focus | 2 Foci | 3 Foci | 4 Foci | 5 Foci | Total (n) |
| --- | --- | --- | --- | --- | --- | --- | --- | --- |
| CYCLING | Cse4 | Expt1 | 1 | 17 | 1 | 0 | 0 | 19 |
| | | Expt2 | 0 | 17 | 2 | 0 | 0 | 19 |
| | | Expt3 | 3 | 17 | 2 | 0 | 0 | 22 |
| | Ndc10 | Expt1 | 4 | 18 | 0 | 0 | 0 | 22 |
| | | Expt2 | 5 | 21 | 2 | 0 | 0 | 28 |
| | | Expt3 | 5 | 18 | 2 | 0 | 0 | 25 |
| ARRESTED | Cse4 | Expt1 | 0 | 24 | 11 | 6 | 1 | 42 |
| | | Expt2 | 0 | 17 | 11 | 1 | 1 | 30 |
| | | Expt3 | 0 | 20 | 7 | 1 | 0 | 28 |
| | Ndc10 | Expt1 | 0 | 17 | 7 | 1 | 1 | 26 |
| | | Expt2 | 0 | 35 | 11 | 1 | 0 | 47 |
| | | Expt3 | 0 | 8 | 2 | 1 | 0 | 11 |
