# Supplementary material for: Nucleolar asymmetry and the importance of septin integrity upon cell cycle arrest
Source: PLoS One. 2017 Mar 24;12(3):e0174306. doi: 10.1371/journal.pone.0174306 (PMC5365125; doi:10.1371/journal.pone.0174306)
Supplement: S4 Table — (PPTX) [file pone.0174306.s012.pptx]

## Slide 1
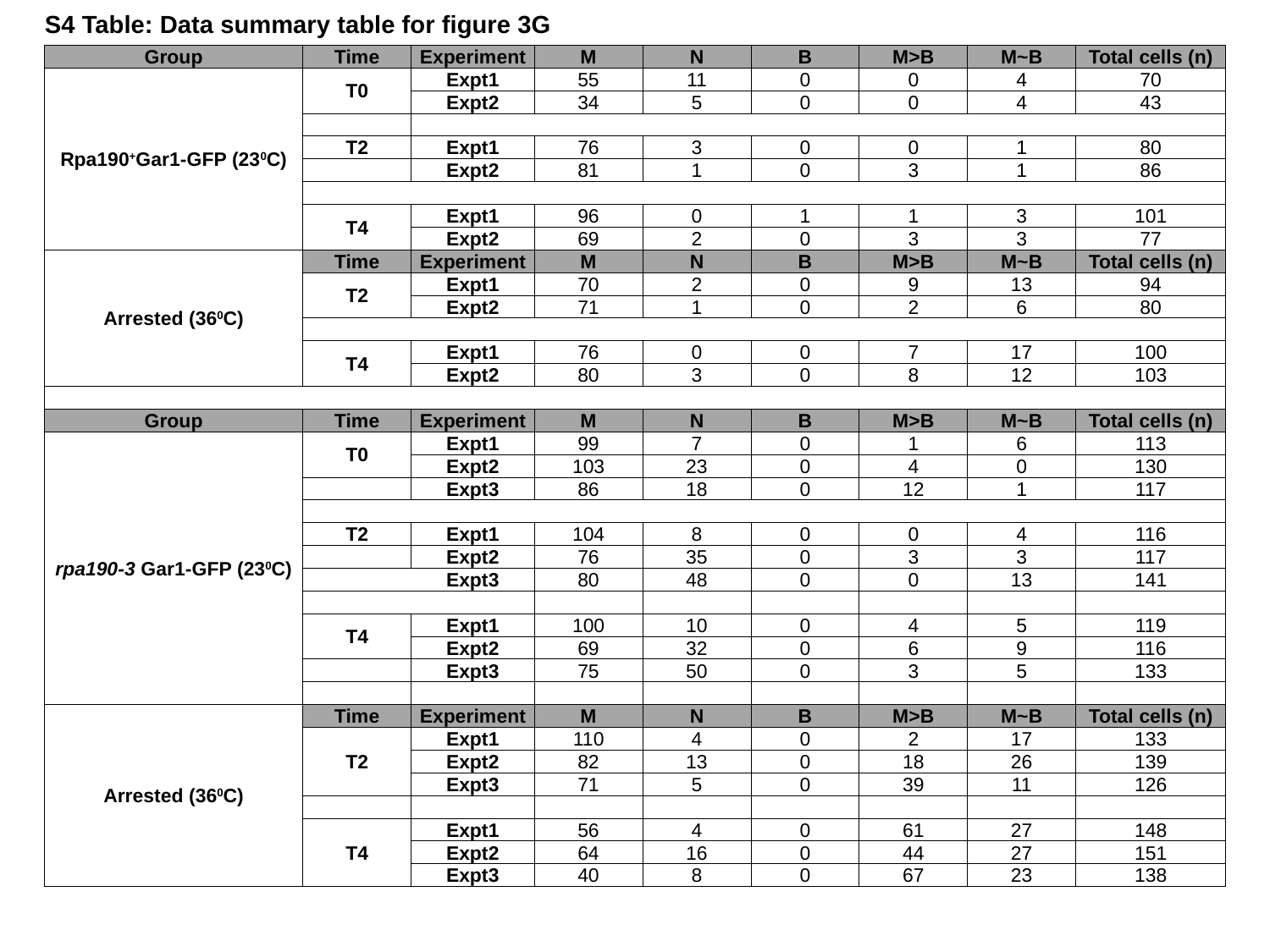

S4 Table: Data summary table for figure 3G
| Group | Time | Experiment | M | N | B | M>B | M~B | Total cells (n) |
| --- | --- | --- | --- | --- | --- | --- | --- | --- |
| Rpa190+Gar1-GFP (230C) | T0 | Expt1 | 55 | 11 | 0 | 0 | 4 | 70 |
| | | Expt2 | 34 | 5 | 0 | 0 | 4 | 43 |
| | | | | | | | | |
| | T2 | Expt1 | 76 | 3 | 0 | 0 | 1 | 80 |
| | | Expt2 | 81 | 1 | 0 | 3 | 1 | 86 |
| | | | | | | | | |
| | T4 | Expt1 | 96 | 0 | 1 | 1 | 3 | 101 |
| | | Expt2 | 69 | 2 | 0 | 3 | 3 | 77 |
| Arrested (360C) | Time | Experiment | M | N | B | M>B | M~B | Total cells (n) |
| | T2 | Expt1 | 70 | 2 | 0 | 9 | 13 | 94 |
| | | Expt2 | 71 | 1 | 0 | 2 | 6 | 80 |
| | | | | | | | | |
| | T4 | Expt1 | 76 | 0 | 0 | 7 | 17 | 100 |
| | | Expt2 | 80 | 3 | 0 | 8 | 12 | 103 |
| | | | | | | | | |
| Group | Time | Experiment | M | N | B | M>B | M~B | Total cells (n) |
| rpa190-3 Gar1-GFP (230C) | T0 | Expt1 | 99 | 7 | 0 | 1 | 6 | 113 |
| | | Expt2 | 103 | 23 | 0 | 4 | 0 | 130 |
| | | Expt3 | 86 | 18 | 0 | 12 | 1 | 117 |
| | | | | | | | | |
| | T2 | Expt1 | 104 | 8 | 0 | 0 | 4 | 116 |
| | | Expt2 | 76 | 35 | 0 | 3 | 3 | 117 |
| | | Expt3 | 80 | 48 | 0 | 0 | 13 | 141 |
| | | | | | | | | |
| | T4 | Expt1 | 100 | 10 | 0 | 4 | 5 | 119 |
| | | Expt2 | 69 | 32 | 0 | 6 | 9 | 116 |
| | | Expt3 | 75 | 50 | 0 | 3 | 5 | 133 |
| | | | | | | | | |
| Arrested (360C) | Time | Experiment | M | N | B | M>B | M~B | Total cells (n) |
| | T2 | Expt1 | 110 | 4 | 0 | 2 | 17 | 133 |
| | | Expt2 | 82 | 13 | 0 | 18 | 26 | 139 |
| | | Expt3 | 71 | 5 | 0 | 39 | 11 | 126 |
| | | | | | | | | |
| | T4 | Expt1 | 56 | 4 | 0 | 61 | 27 | 148 |
| | | Expt2 | 64 | 16 | 0 | 44 | 27 | 151 |
| | | Expt3 | 40 | 8 | 0 | 67 | 23 | 138 |
