# Supplementary material for: Nucleolar asymmetry and the importance of septin integrity upon cell cycle arrest
Source: PLoS One. 2017 Mar 24;12(3):e0174306. doi: 10.1371/journal.pone.0174306 (PMC5365125; doi:10.1371/journal.pone.0174306)
Supplement: S5 Table — (PPTX) [file pone.0174306.s013.pptx]

## Slide 1
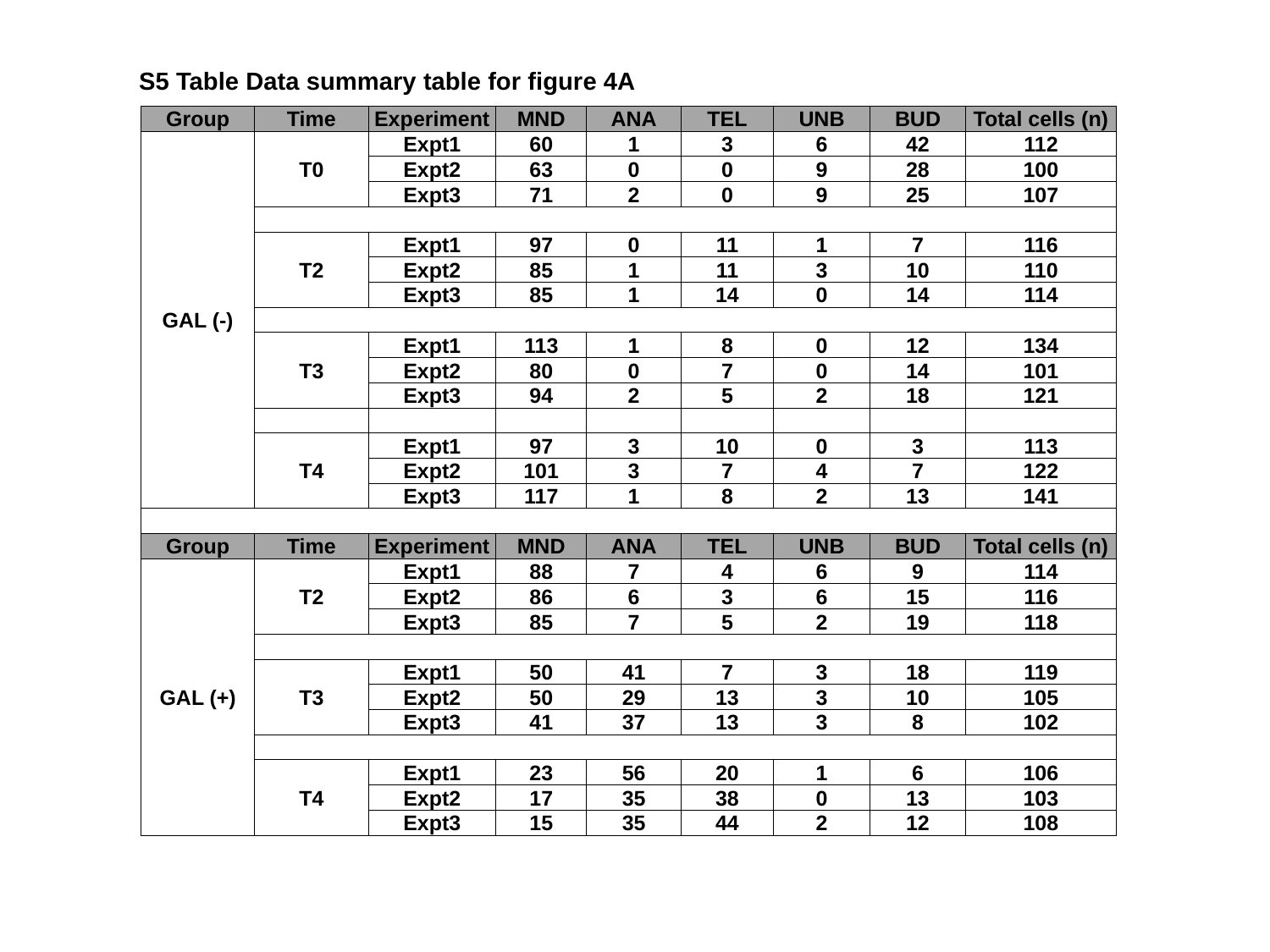

S5 Table Data summary table for figure 4A
| Group | Time | Experiment | MND | ANA | TEL | UNB | BUD | Total cells (n) |
| --- | --- | --- | --- | --- | --- | --- | --- | --- |
| GAL (-) | T0 | Expt1 | 60 | 1 | 3 | 6 | 42 | 112 |
| | | Expt2 | 63 | 0 | 0 | 9 | 28 | 100 |
| | | Expt3 | 71 | 2 | 0 | 9 | 25 | 107 |
| | | | | | | | | |
| | T2 | Expt1 | 97 | 0 | 11 | 1 | 7 | 116 |
| | | Expt2 | 85 | 1 | 11 | 3 | 10 | 110 |
| | | Expt3 | 85 | 1 | 14 | 0 | 14 | 114 |
| | | | | | | | | |
| | T3 | Expt1 | 113 | 1 | 8 | 0 | 12 | 134 |
| | | Expt2 | 80 | 0 | 7 | 0 | 14 | 101 |
| | | Expt3 | 94 | 2 | 5 | 2 | 18 | 121 |
| | | | | | | | | |
| | T4 | Expt1 | 97 | 3 | 10 | 0 | 3 | 113 |
| | | Expt2 | 101 | 3 | 7 | 4 | 7 | 122 |
| | | Expt3 | 117 | 1 | 8 | 2 | 13 | 141 |
| | | | | | | | | |
| Group | Time | Experiment | MND | ANA | TEL | UNB | BUD | Total cells (n) |
| GAL (+) | T2 | Expt1 | 88 | 7 | 4 | 6 | 9 | 114 |
| | | Expt2 | 86 | 6 | 3 | 6 | 15 | 116 |
| | | Expt3 | 85 | 7 | 5 | 2 | 19 | 118 |
| | | | | | | | | |
| | T3 | Expt1 | 50 | 41 | 7 | 3 | 18 | 119 |
| | | Expt2 | 50 | 29 | 13 | 3 | 10 | 105 |
| | | Expt3 | 41 | 37 | 13 | 3 | 8 | 102 |
| | | | | | | | | |
| | T4 | Expt1 | 23 | 56 | 20 | 1 | 6 | 106 |
| | | Expt2 | 17 | 35 | 38 | 0 | 13 | 103 |
| | | Expt3 | 15 | 35 | 44 | 2 | 12 | 108 |
