# Supplementary material for: Nucleolar asymmetry and the importance of septin integrity upon cell cycle arrest
Source: PLoS One. 2017 Mar 24;12(3):e0174306. doi: 10.1371/journal.pone.0174306 (PMC5365125; doi:10.1371/journal.pone.0174306)
Supplement: S6 Table — (PPTX) [file pone.0174306.s014.pptx]

## Slide 1
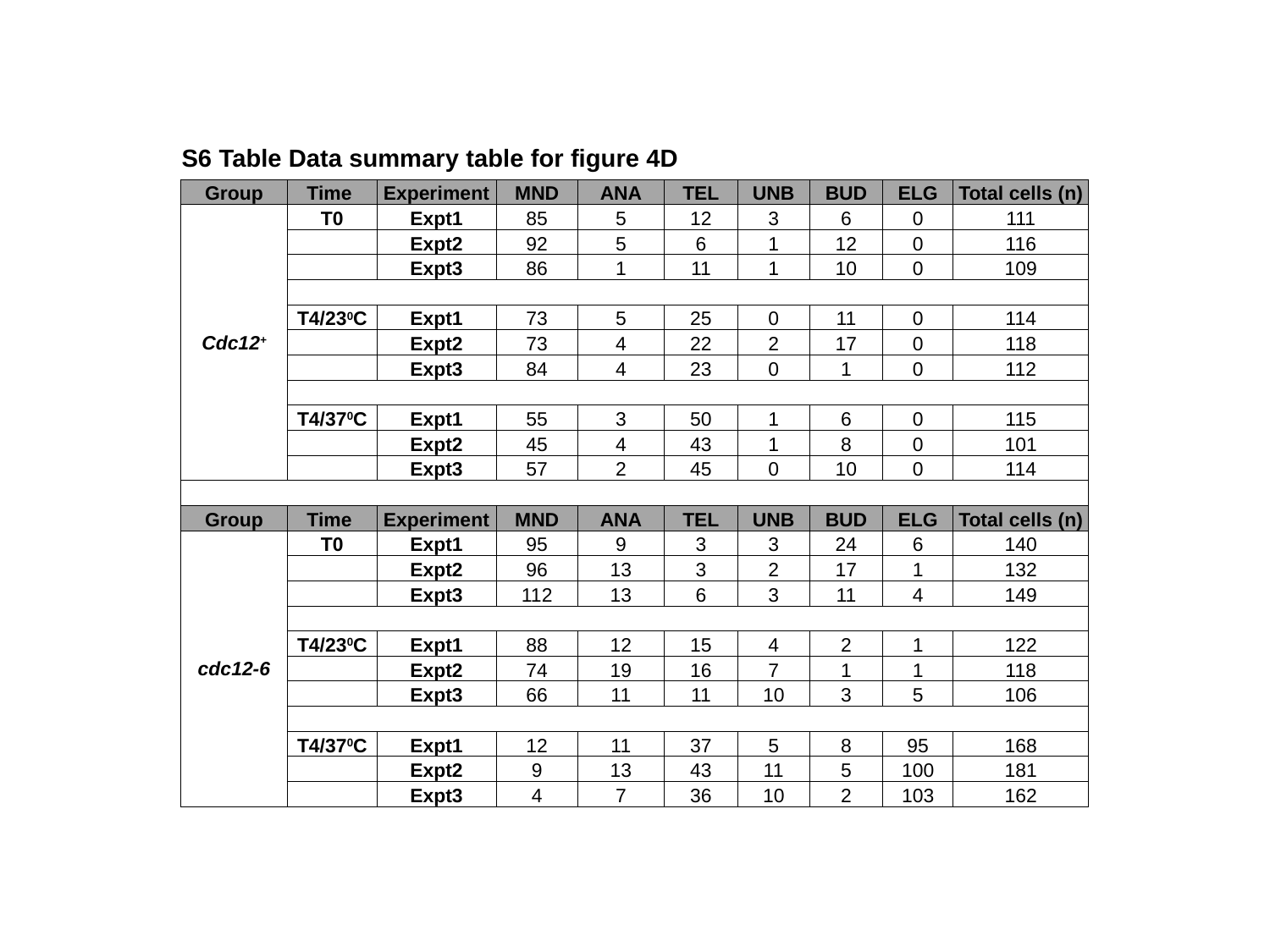

S6 Table Data summary table for figure 4D
| Group | Time | Experiment | MND | ANA | TEL | UNB | BUD | ELG | Total cells (n) |
| --- | --- | --- | --- | --- | --- | --- | --- | --- | --- |
| Cdc12+ | T0 | Expt1 | 85 | 5 | 12 | 3 | 6 | 0 | 111 |
| | | Expt2 | 92 | 5 | 6 | 1 | 12 | 0 | 116 |
| | | Expt3 | 86 | 1 | 11 | 1 | 10 | 0 | 109 |
| | | | | | | | | | |
| | T4/230C | Expt1 | 73 | 5 | 25 | 0 | 11 | 0 | 114 |
| | | Expt2 | 73 | 4 | 22 | 2 | 17 | 0 | 118 |
| | | Expt3 | 84 | 4 | 23 | 0 | 1 | 0 | 112 |
| | | | | | | | | | |
| | T4/370C | Expt1 | 55 | 3 | 50 | 1 | 6 | 0 | 115 |
| | | Expt2 | 45 | 4 | 43 | 1 | 8 | 0 | 101 |
| | | Expt3 | 57 | 2 | 45 | 0 | 10 | 0 | 114 |
| | | | | | | | | | |
| Group | Time | Experiment | MND | ANA | TEL | UNB | BUD | ELG | Total cells (n) |
| cdc12-6 | T0 | Expt1 | 95 | 9 | 3 | 3 | 24 | 6 | 140 |
| | | Expt2 | 96 | 13 | 3 | 2 | 17 | 1 | 132 |
| | | Expt3 | 112 | 13 | 6 | 3 | 11 | 4 | 149 |
| | | | | | | | | | |
| | T4/230C | Expt1 | 88 | 12 | 15 | 4 | 2 | 1 | 122 |
| | | Expt2 | 74 | 19 | 16 | 7 | 1 | 1 | 118 |
| | | Expt3 | 66 | 11 | 11 | 10 | 3 | 5 | 106 |
| | | | | | | | | | |
| | T4/370C | Expt1 | 12 | 11 | 37 | 5 | 8 | 95 | 168 |
| | | Expt2 | 9 | 13 | 43 | 11 | 5 | 100 | 181 |
| | | Expt3 | 4 | 7 | 36 | 10 | 2 | 103 | 162 |
