# Supplementary material for: Nucleolar asymmetry and the importance of septin integrity upon cell cycle arrest
Source: PLoS One. 2017 Mar 24;12(3):e0174306. doi: 10.1371/journal.pone.0174306 (PMC5365125; doi:10.1371/journal.pone.0174306)
Supplement: S7 Table — (PPTX) [file pone.0174306.s015.pptx]

## Slide 1
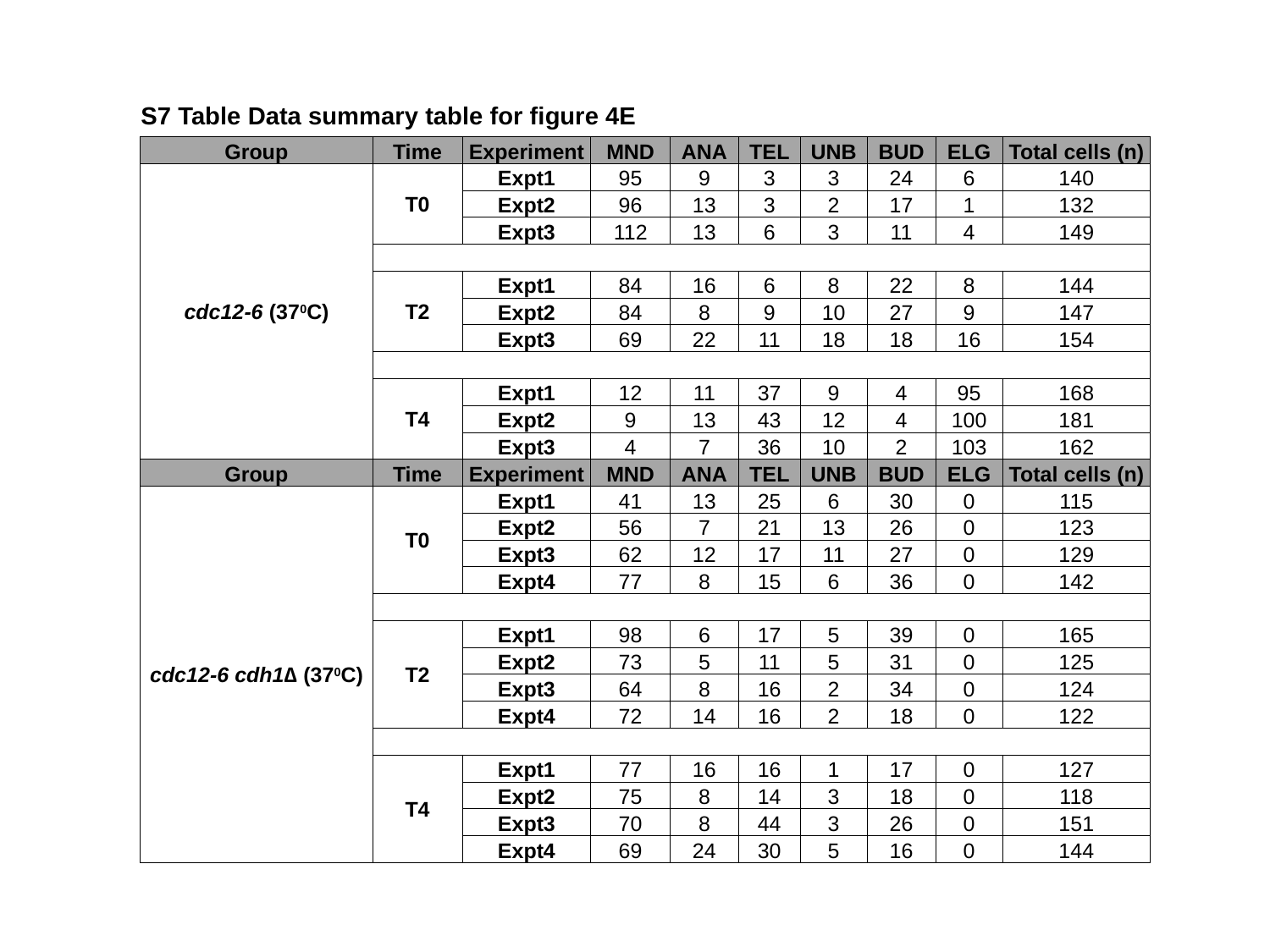

S7 Table Data summary table for figure 4E
| Group | Time | Experiment | MND | ANA | TEL | UNB | BUD | ELG | Total cells (n) |
| --- | --- | --- | --- | --- | --- | --- | --- | --- | --- |
| cdc12-6 (370C) | T0 | Expt1 | 95 | 9 | 3 | 3 | 24 | 6 | 140 |
| | | Expt2 | 96 | 13 | 3 | 2 | 17 | 1 | 132 |
| | | Expt3 | 112 | 13 | 6 | 3 | 11 | 4 | 149 |
| | | | | | | | | | |
| | T2 | Expt1 | 84 | 16 | 6 | 8 | 22 | 8 | 144 |
| | | Expt2 | 84 | 8 | 9 | 10 | 27 | 9 | 147 |
| | | Expt3 | 69 | 22 | 11 | 18 | 18 | 16 | 154 |
| | | | | | | | | | |
| | T4 | Expt1 | 12 | 11 | 37 | 9 | 4 | 95 | 168 |
| | | Expt2 | 9 | 13 | 43 | 12 | 4 | 100 | 181 |
| | | Expt3 | 4 | 7 | 36 | 10 | 2 | 103 | 162 |
| Group | Time | Experiment | MND | ANA | TEL | UNB | BUD | ELG | Total cells (n) |
| cdc12-6 cdh1∆ (370C) | T0 | Expt1 | 41 | 13 | 25 | 6 | 30 | 0 | 115 |
| | | Expt2 | 56 | 7 | 21 | 13 | 26 | 0 | 123 |
| | | Expt3 | 62 | 12 | 17 | 11 | 27 | 0 | 129 |
| | | Expt4 | 77 | 8 | 15 | 6 | 36 | 0 | 142 |
| | | | | | | | | | |
| | T2 | Expt1 | 98 | 6 | 17 | 5 | 39 | 0 | 165 |
| | | Expt2 | 73 | 5 | 11 | 5 | 31 | 0 | 125 |
| | | Expt3 | 64 | 8 | 16 | 2 | 34 | 0 | 124 |
| | | Expt4 | 72 | 14 | 16 | 2 | 18 | 0 | 122 |
| | | | | | | | | | |
| | T4 | Expt1 | 77 | 16 | 16 | 1 | 17 | 0 | 127 |
| | | Expt2 | 75 | 8 | 14 | 3 | 18 | 0 | 118 |
| | | Expt3 | 70 | 8 | 44 | 3 | 26 | 0 | 151 |
| | | Expt4 | 69 | 24 | 30 | 5 | 16 | 0 | 144 |
