# Supplementary material for: Nucleolar asymmetry and the importance of septin integrity upon cell cycle arrest
Source: PLoS One. 2017 Mar 24;12(3):e0174306. doi: 10.1371/journal.pone.0174306 (PMC5365125; doi:10.1371/journal.pone.0174306)
Supplement: S8 Table — (PPTX) [file pone.0174306.s016.pptx]

## Slide 1
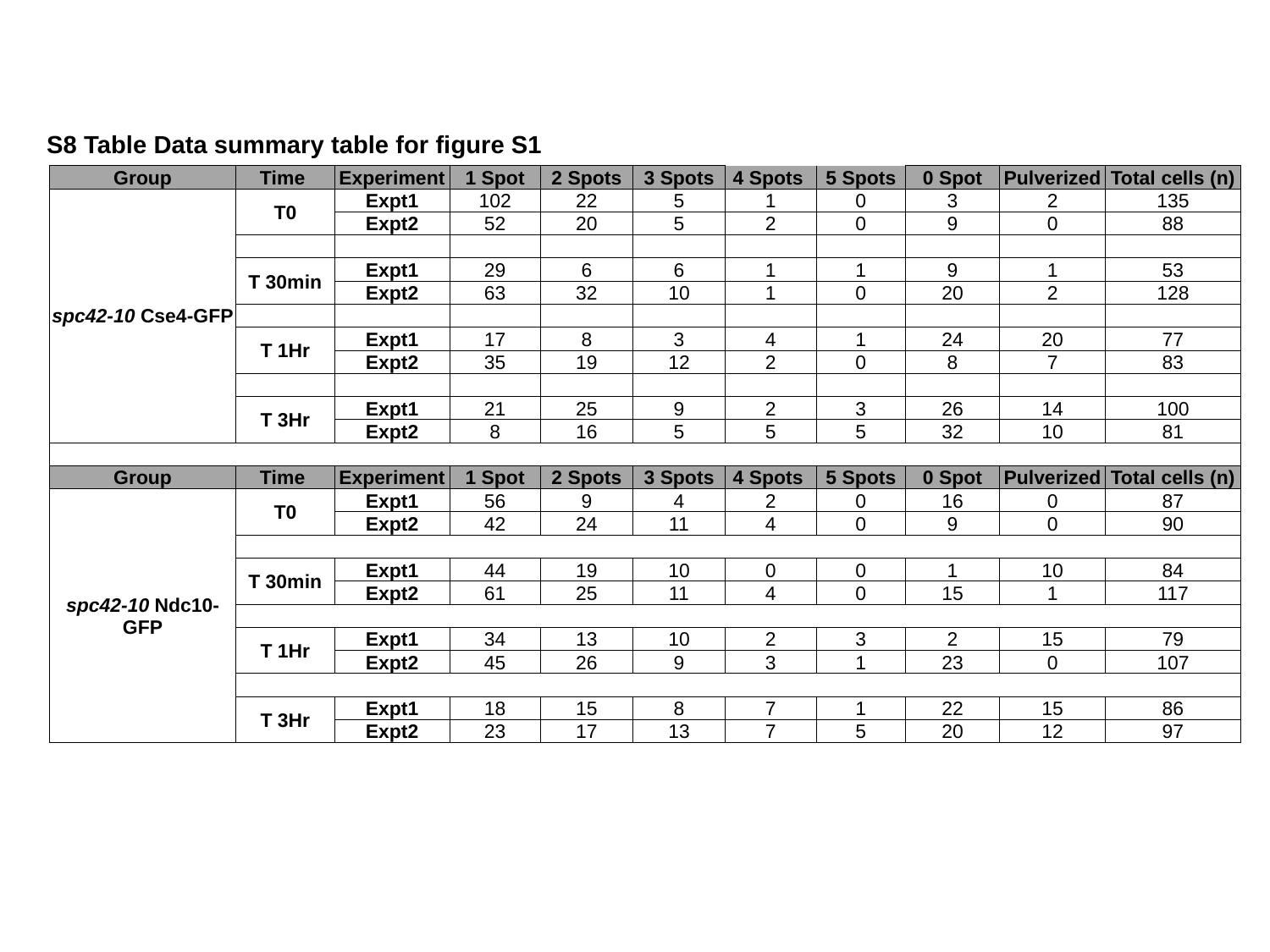

S8 Table Data summary table for figure S1
| Group | Time | Experiment | 1 Spot | 2 Spots | 3 Spots | 4 Spots | 5 Spots | 0 Spot | Pulverized | Total cells (n) |
| --- | --- | --- | --- | --- | --- | --- | --- | --- | --- | --- |
| spc42-10 Cse4-GFP | T0 | Expt1 | 102 | 22 | 5 | 1 | 0 | 3 | 2 | 135 |
| | | Expt2 | 52 | 20 | 5 | 2 | 0 | 9 | 0 | 88 |
| | | | | | | | | | | |
| | T 30min | Expt1 | 29 | 6 | 6 | 1 | 1 | 9 | 1 | 53 |
| | | Expt2 | 63 | 32 | 10 | 1 | 0 | 20 | 2 | 128 |
| | | | | | | | | | | |
| | T 1Hr | Expt1 | 17 | 8 | 3 | 4 | 1 | 24 | 20 | 77 |
| | | Expt2 | 35 | 19 | 12 | 2 | 0 | 8 | 7 | 83 |
| | | | | | | | | | | |
| | T 3Hr | Expt1 | 21 | 25 | 9 | 2 | 3 | 26 | 14 | 100 |
| | | Expt2 | 8 | 16 | 5 | 5 | 5 | 32 | 10 | 81 |
| | | | | | | | | | | |
| Group | Time | Experiment | 1 Spot | 2 Spots | 3 Spots | 4 Spots | 5 Spots | 0 Spot | Pulverized | Total cells (n) |
| spc42-10 Ndc10-GFP | T0 | Expt1 | 56 | 9 | 4 | 2 | 0 | 16 | 0 | 87 |
| | | Expt2 | 42 | 24 | 11 | 4 | 0 | 9 | 0 | 90 |
| | | | | | | | | | | |
| | T 30min | Expt1 | 44 | 19 | 10 | 0 | 0 | 1 | 10 | 84 |
| | | Expt2 | 61 | 25 | 11 | 4 | 0 | 15 | 1 | 117 |
| | | | | | | | | | | |
| | T 1Hr | Expt1 | 34 | 13 | 10 | 2 | 3 | 2 | 15 | 79 |
| | | Expt2 | 45 | 26 | 9 | 3 | 1 | 23 | 0 | 107 |
| | | | | | | | | | | |
| | T 3Hr | Expt1 | 18 | 15 | 8 | 7 | 1 | 22 | 15 | 86 |
| | | Expt2 | 23 | 17 | 13 | 7 | 5 | 20 | 12 | 97 |
